# AZD5582 plus SIV-specific antibodies reduce lymph node viral reservoirs in antiretroviral therapy-suppressed macaques

**DOI:** 10.1038/s41591-023-02570-7

**Published:** 2023-10-02

**Authors:** Amir Dashti, Sophia Sukkestad, Anna M. Horner, Margaret Neja, Zain Siddiqi, Chevaughn Waller, Jordan Goldy, Dominique Monroe, Alice Lin, Nils Schoof, Vidisha Singh, Maud Mavigner, Jeffrey D. Lifson, Claire Deleage, Marina Tuyishime, Shane D. Falcinelli, Hannah A. D. King, Ruian Ke, Rosemarie D. Mason, Nancie M. Archin, Richard M. Dunham, Jeffrey T. Safrit, Sherrie Jean, Alan S. Perelson, David M. Margolis, Guido Ferrari, Mario Roederer, Guido Silvestri, Ann Chahroudi

**Affiliations:** 1 Department of Pediatrics, Emory University School of Medicine, Atlanta, GA USA; 2 Emory National Primate Research Center, Emory University, Atlanta, GA USA; 3 Center for Childhood Infections and Vaccines of Children’s Healthcare of Atlanta and Emory University, Atlanta, GA USA; 4 AIDS and Cancer Virus Program, Frederick National Laboratory for Cancer Research, Frederick, MD USA; 5 Department of Surgery, Duke University Medical Center, Durham, NC USA; 6 UNC HIV Cure Center and Department of Medicine, University of North Carolina at Chapel Hill, Chapel Hill, NC USA; 7 Vaccine Research Center, National Institute of Allergy and Infectious Disease, National Institutes of Health, Bethesda, MD USA; 8 US Military HIV Research Program, Walter Reed Army Institute of Research, Silver Spring, MD USA; 9 Henry M. Jackson Foundation for the Advancement of Military Medicine, Bethesda, MD USA; 10 Theoretical Biology and Biophysics Group, Los Alamos National Laboratory, Los Alamos, NM USA; 11 HIV Drug Discovery, ViiV Healthcare, Research Traingle Park, NC USA; 12 ImmunityBio, Inc., Culver City, CA USA; 13 Duke Human Vaccine Institute, Duke University Medical Center, Durham, NC USA

**Keywords:** Pharmaceutics, HIV infections

## Abstract

The main barrier to HIV cure is a persistent reservoir of latently infected CD4^+^ T cells harboring replication-competent provirus that fuels rebound viremia upon antiretroviral therapy (ART) interruption. A leading approach to target this reservoir involves agents that reactivate latent HIV proviruses followed by direct clearance of cells expressing induced viral antigens by immune effector cells and immunotherapeutics. We previously showed that AZD5582, an antagonist of inhibitor of apoptosis proteins and mimetic of the second mitochondrial-derived activator of caspases (IAPi/SMACm), induces systemic reversal of HIV/SIV latency but with no reduction in size of the viral reservoir. In this study, we investigated the effects of AZD5582 in combination with four SIV Env-specific Rhesus monoclonal antibodies (RhmAbs) ± N-803 (an IL-15 superagonist) in SIV-infected, ART-suppressed rhesus macaques. Here we confirm the efficacy of AZD5582 in inducing SIV reactivation, demonstrate enhancement of latency reversal when AZD5582 is used in combination with N-803 and show a reduction in total and replication-competent SIV-DNA in lymph-node-derived CD4^+^ T cells in macaques treated with AZD5582 + RhmAbs. Further exploration of this therapeutic approach may contribute to the goal of achieving an HIV cure.

## Abstract (web summary)

Reactivation of latent SIV with an IAP antagonist, coupled with immunotherapeutic approaches, reduced replication-competent SIV in lymph node CD4^+^ T cells in rhesus macaques. Further exploration targeting these pathways to reduce the HIV viral reservoir is warranted.

## Main

The reservoir of HIV provirus that persists despite antiretroviral therapy (ART) and can initiate viral rebound when ART is stopped is the greatest obstacle to an HIV cure^1,2^. Accordingly, interruption of ART almost invariably leads to viral rebound^3^. Among HIV cure strategies^4–7^, the latency reversal and clearance approach aims to reactivate viral expression in persistently infected cells followed by immune-mediated clearance of these newly exposed targets, reducing or potentially eliminating the HIV reservoir^8–18^. We previously showed that AZD5582, an inhibitor of inhibitor of apoptosis proteins (IAPi) and mimetic of the second mitochondrial-derived activator of caspases (SMACm) induces systemic latency reversal in vivo in SIV-infected, ART-suppressed rhesus macaques (RMs) and HIV-infected, ART-treated humanized mice^16^. This latency reversal was characterized by the onset of viremia despite ART, measurable with standard viral load assays and increased viral RNA in tissues. Despite sustained virus reactivation, we did not find a consistent reduction in the size of the viral reservoir with AZD5582 treatment alone. These findings are consistent with clinical studies of latency reversal agents (LRAs) and indicate that augmentation of immune effector mechanisms will be necessary to clear reactivated, infected cells.

In the current study, we sought to eliminate virally infected cells that persist during ART by combining AZD5582 with four SIV-specific Rhesus monoclonal antibodies (RhmAbs) to target viral Env-expressing cells for immune-mediated clearance. To maximize recognition, we selected RhmAbs that each target a distinct region of the SIV Env, and included both SIV_mac239_ neutralizing and non-neutralizing mAbs^19,20^. We also sought to enhance immune effector function and clearance of cells expressing induced viral antigens through the addition of the IL-15 superagonist N-803 in a subset of animals. N-803 has been shown to increase the proliferation and trafficking of CD8^+^ T cells and natural killer (NK) cells to lymph nodes^19,21^, a major site of HIV/SIV persistence^22^. As N-803 has also been reported to reverse latency in some studies^16,17,23^, we also hypothesized that the dual LRA combination of AZD5582 and N-803 might enhance virus reactivation in latently infected cells.

## Results

### Study design and intervention strategy

To evaluate the combination of an IAPi/SMACm ± IL-15 superagonist and SIV Env-targeting RhmAbs to reduce the established viral reservoir, we used a macaque model of SIV_mac239_ infection with ART-mediated suppression of viral replication. SIV-specific RhmAbs were isolated from SIV-infected Indian RMs and expressed as full-length rhesus IgG1 modified to contain the LS-encoding mutation (M428L/N434S) to maximize circulation half-life. The selected RhmAbs—ITS09.01-LS, ITS102.01-LS, ITS103.01-LS and ITS113.01-LS—are specific for the V2, CD4bs, CD4bs proximal and membrane-proximal external region (MPER) of SIV Env, respectively. Neutralization activity for the highly neutralization resistant (tier-3) SIV_mac239_ has been characterized, with ITS09.01-LS being the only non-neutralizer^19–21^. We hypothesized that SIV Env RhmAbs would enhance the elimination of latently infected CD4^+^ T cells through mechanisms other than neutralization of free virions, such as antibody-dependent cellular cytotoxicity (ADCC). The IL-15 superagonist N-803 was added to RhmAb + AZD5582 combination therapy in a subset of animals to promote NK cell proliferation and lymph node trafficking^24^.

**Fig. 1:**
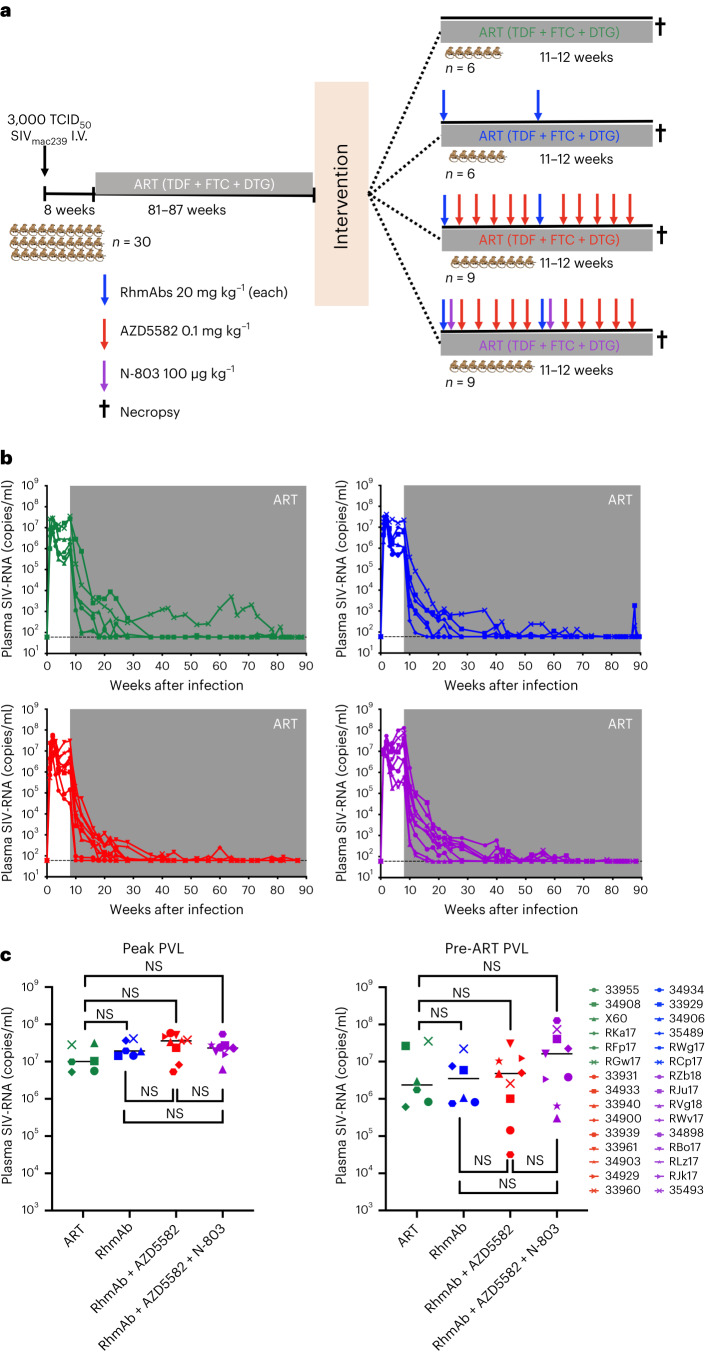
Study design and animal group assignment. **a**, Experimental design. Thirty RMs were infected with SIV_mac239_. ART was initiated 8 weeks p.i. After 81–87 weeks of ART, RMs were divided into four groups with continuous ART exposure: ART controls (*n* = 6); RhmAb controls (*n* = 6); RhmAb + AZD5582 (*n* = 9); and RhmAb + AZD5582 + N-803 (*n* = 9). RhmAbs were dosed twice at 20 mg kg^−1^ s.c. each; AZD5582 at 0.1 mg kg^−1^ i.v. weekly for 10 weeks and N-803 at 100 μg kg^−1^ s.c. twice as shown. The first RhmAbs injection was administered 3 d before the first dose of AZD5582. N-803 was given at the same time as RhmAbs. **b**, Plasma SIV-RNA levels in the 30 RMs after SIV infection and ART but before further interventions. Green symbols: ART control group (*n* = 6); blue symbols: RhmAb control group (*n* = 6); red symbols: RhmAb + AZD5582 group (*n* = 9); purple symbols: RhmAb + AZD5582 + N-803 group (*n* = 9). Gray shading represents the period of ART administration **c**, Peak and pre-ART viral loads compared across groups: ART controls (*n* = 6); RhmAb controls (*n* = 6); RhmAb + AZD5582 (*n* = 9); and RhmAb + AZD5582 + N-803 (*n* = 9). Statistical significance was determined with a two-sided Mann–Whitney *U*-test. Horizontal lines represent the median.

In total, 30 Indian-origin RMs (25 males and five females, all negative for the Mamu-B*08 and Mamu-B*17 major histocompatibility complex (MHC) class I alleles associated with an increased frequency of spontaneous control of SIV replication) were infected with SIV_mac239_ by a single intravenous (i.v.) administration of 3,000 50% tissue culture infective doses (TCID_50_) (Fig. 1a). ART consisting of tenofovir (TDF), emtricitabine (FTC) and dolutegravir (DTG) was administered daily by subcutaneous (s.c.) injection from week 8 post-infection (p.i.) to the end of the study. This ART regimen effectively suppressed SIV-RNA in plasma below the limit of detection (LOD) of the standard plasma viral load (PVL) assay used (60 copies per milliliter) in most animals (Fig. 1b). Time to viral suppression was variable, with a mean of 32 weeks to first undetectable value (<60 copies per milliliter), followed by some RMs showing transient blips of viremia. PVLs were also measured by ultrasensitive assay with a lower detection limit (<4 copies per milliliter) at week 71 p.i., revealing a median of 10 RNA copies per milliliter (range, 4–33 RNA copies per milliliter), thus demonstrating this model’s excellent approximation of people living with HIV on long-term ART.

After 81–87 weeks of ART and before additional interventions, groups were balanced for peak viral loads and pre-ART viral loads (Fig. 1c). Sex, Mamu-A*01 status and age at infection were also considered (Table 1). Groups were then treated as follows: (1) six RMs did not receive interventional treatment (ART control); (2) six RMs received two doses of the four-RhmAbs cocktail (20 mg kg^−1^ s.c. each) 5 weeks apart (RhmAb control); (3) nine RMs received two doses of the four-RhmAbs cocktail (20 mg kg^−1^ s.c. each) 5 weeks apart with 10 weekly doses of AZD5582 (0.1 mg kg^−1^ i.v.) (RhmAb + AZD5582); and (4) nine RMs received two doses of both the cocktail of four RhmAbs (20 mg kg^−1^ s.c. each) and N-803 (100 μg kg^−1^ s.c.) 5 weeks apart with 10 weekly doses of AZD5582 (0.1 mg kg^−1^ i.v.) (RhmAb + AZD5582 + N-803) (Fig. 1a). All animals received ART until euthanasia and necropsy.

**Table 1:** Characteristics of Study Groups

Group	ID	Sex	A01 status	Age at infection, years	Peak PVL	Pre-ART PVL
ART	33955	Male	+	4.03	5.61×10^6^	8.32×10^5^
	34908	Male	−	3.00	1.03×10^7^	2.65×10^7^
	X60	Female	−	4.10	3.18×10^7^	2.99×10^6^
	RKa17	Male	−	2.98	5.23×10^6^	6.07×10^5^
	RFp17	Male	−	2.86	9.82×10^6^	1.75×10^6^
	RGw17	Male	−	2.86	2.84×10^7^	3.54×10^7^
RhmAb	34934	Male	+	3.00	1.44×10^7^	8.13×10^5^
	33929	Male	−	4.04	1.43×10^7^	5.94×10^6^
	34906	Male	−	3.01	1.91×10^7^	1.07×10^6^
	35489	Female	−	2.89	3.68×10^7^	7.57×10^6^
	RWg17	Male	−	3.83	1.95×10^7^	7.50×10^5^
	RCp17	Male	−	2.99	4.12×10^7^	2.20×10^7^
RhmAb + AZD5582	33931	Male	+	4.04	5.80×10^7^	1.43×10^5^
	34933	Male	−	3.00	2.36×10^7^	1.01×10^6^
	33940	Male	−	4.04	3.38×10^7^	4.80×10^6^
	34900	Male	−	3.19	8.19×10^6^	4.98×10^6^
	33939	Male	−	4.54	5.31×10^6^	3.17×10^4^
	33961	Male	−	3.96	5.12×10^7^	3.03×10^7^
	34903	Male	−	3.18	3.62×10^7^	1.04×10^7^
	34929	Male	−	3.42	4.60×10^7^	1.20×10^7^
	33960	Male	−	3.87	3.80×10^7^	2.59×10^6^
RhmAb + AZD5582 + N-803	RZb18	Male	−	2.78	2.40×10^7^	3.83×10^6^
	RJu17	Male	−	2.88	2.72×10^7^	4.08×10^7^
	RVg18	Male	−	2.78	6.21×10^6^	3.04×10^5^
	RWv17	Male	−	2.87	2.26×10^7^	2.24×10^7^
	34898	Male	−	3.58	5.46×10^7^	1.28×10^8^
	RBo17	Female	−	3.59	1.85×10^7^	1.62×10^7^
	RLz17	Male	−	2.82	2.80×10^7^	6.44×10^5^
	RJk17	Female	−	3.77	1.52×10^7^	3.32×10^6^
	35493	Female	−	2.88	2.32×10^7^	7.36×10^7^

### SIV Env-specific RhmAbs characteristics and function

**Fig. 2:**
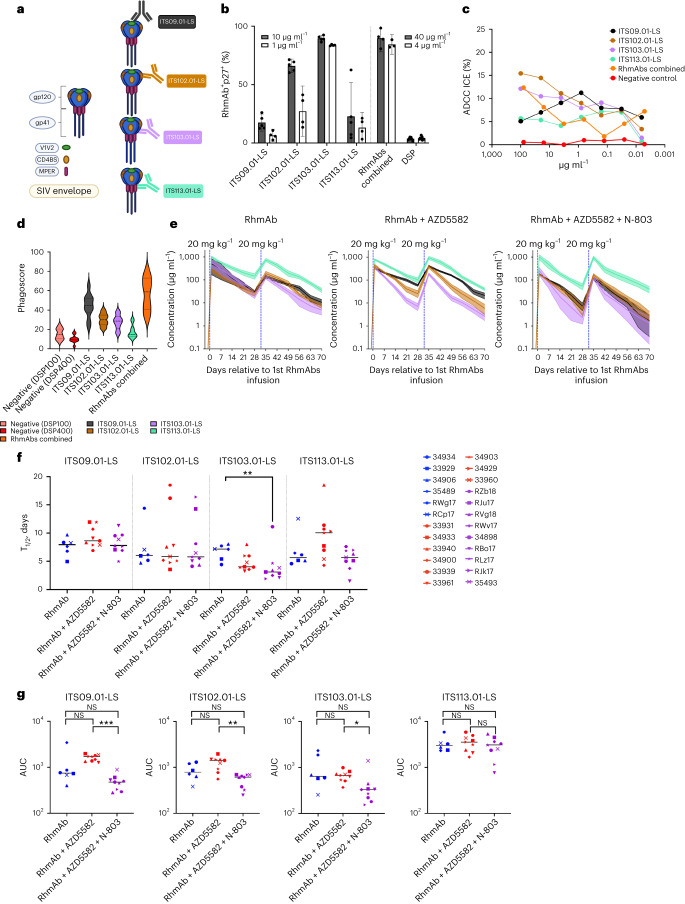
RhmAb specificities and function. **a**, Schematic representation of RhmAbs used in this study. ITS09.01-LS, ITS102.01-LS, ITS103.01-LS and ITS113.01-LS RhmAbs target V2, CD4 binding site, CD4 binding site proximal and MPER, respectively. **b**, Surface binding of RhmAbs to SIV_mac239_-infected A66 cells. The percentage of infected cells (p27^+^) with bound RhmAbs (at 10 µg ml^−1^ and 1 μg ml^−1^) was measured by flow cytometry. Combo indicates the binding of a combination of all four RhmAbs (total of 40 µg ml^−1^ and 4 μg ml^−1^). Anti-DSP was used as a negative control at both 40 µg ml^−1^ and 4 µg ml^−1^. Technical replicates are shown: *n* = 5 (10 µg ml^−1^); *n* = 4 (1 μg ml^−1^ and combo at 10 µg ml^−1^ each); *n* = 3 (combo at 1 μg ml^−1^ each); *n* = 7 (DSP 40 µg ml^−1^); *n* = 9 (DSP 4 µg ml^−1^). Mean with 95% confidence intervals are shown. **c**, In vitro analysis of ICE ADCC assay using SIV_mac239_-infected or mock-infected A66 cells as targets and NK92RhCD16 cells as effectors at a 10:1 effector:target ratio. Individual RhmAbs were tested using five-fold dilutions starting at 100 µg ml^−1^ concentration. The cocktail of four RhmAbs was also tested starting with 80 µg ml^−1^ total IgG (20 µg ml^−1^ of each RhmAb). The negative control DSP was tested using five-fold dilutions starting at 80 µg ml^−1^. **d**, In vitro analysis of ADNP using granulocytes isolated from PBMCs of SIV_mac239_-infected RMs as effectors and SIV_mac239_ gp130-coated fluorescent beads as targets. Individual RhmAbs were tested using 100 µg ml^−1^ concentration. Combined RhmAbs were tested using 100 µg ml^−1^ concentration of each RhmAb. The negative control DSP was tested at 100 µg ml^−1^ and 400 µg ml^−1^ (DSP100 and DSP400). **e**, RhmAb concentrations in serum (mean ± s.e.m.) are shown after each of the two injections of RhmAbs over10 weeks of the intervention phase of the experiment. Vertical blue lines show the timing of RhmAb administration. **f**, Half-life (T_1/2_) of RhmAbs after the first dose. RhmAb controls (*n* = 6), RhmAb + AZD5582 (*n* = 9) and RhmAb + AZD5582 + N-803 (*n* = 9). **g**, AUC of RhmAb concentrations from 24 h after the first dose through day 70. RhmAb controls (*n* = 6), RhmAb + AZD5582 (*n* = 9) and RhmAb + AZD5582 + N-803 (*n* = 9). Statistical significance was determined using the Kruskal–Wallis test with Dunn’s multiple comparisons test in **f** and **g**. Horizontal lines represent the median. NS, non-significant. *P < 0.05; **P < 0.01; ***P < 0.001.

**Extended Data Fig. 1:**
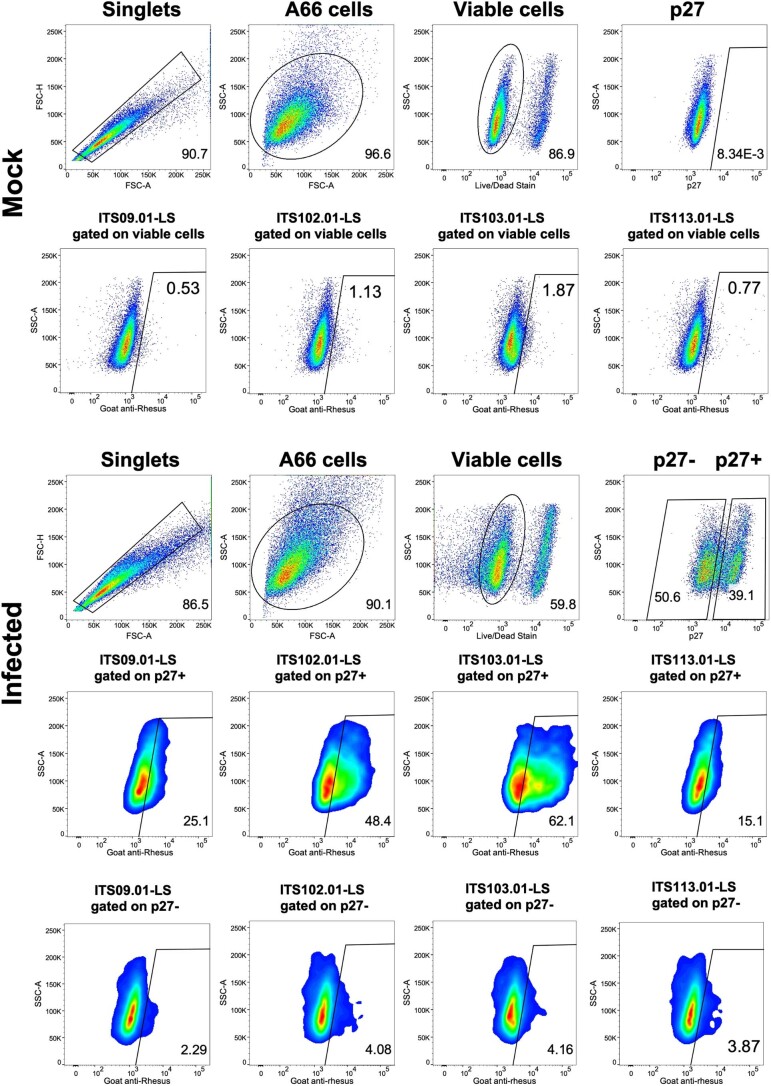
Gating strategy for flow cytometric analysis of RhmAb binding to infected cells. Gating strategy for flow cytometric analysis of RhmAb binding to mock-infected A66 cell line, expressing rhesus CD4 and CCR5 receptors (top) and SIV_mac239_-infected A66 cells (bottom). For SIV_mac239_-infected cells, p27^+^ and p27^−^ cells were analyzed for surface binding by staining with secondary Ab (goat anti-human (H + L) polyclonal antisera) after incubation with RhmAbs.

Before in vivo administration of the selected SIV RhmAbs (Fig. 2a), we assessed their functional ability in vitro. The infected cell antibody binding assay (ICABA) was used to measure the percentage of infected cells (SIV p27^+^) with surface-bound SIV RhmAbs. Specificity of binding was assessed in both mock-infected and SIV_mac239_-exposed p27^−^ cells with less than 5% positivity observed for each RhmAb (see Extended Data Fig. 1 for representative gating strategy). Using 10 µg ml^−1^ of ITS09.01-LS, ITS102.01-LS, ITS103.01-LS and ITS113.01-LS resulted in 17.44 ± 2.63%, 66.04 ± 2.11%, 89.54 ± 1.02% and 22.81 ± 10.45% p27^+^RhmAb^+^ cells, respectively (mean ± s.e.m.) (Fig. 2b). The combination of all four RhmAbs was similar to that of the highest binding RhmAb alone (ITS103.01-LS) with 89.72 ± 3.66% and 84.46 ± 1.98% for 40 µg ml^−1^ and 4 µg ml^−1^, respectively (Fig. 2b). The infected cell elimination (ICE) assay^25^ was used to assess ADCC, with ITS102.01-LS demonstrating the greatest ADCC at the two highest concentrations (15.4% and 14.4%, respectively), followed by ITS103.01-LS and the four RhmAbs combined (Fig. 2c). ITS09.01-LS showed a prozone phenomenon (where excess antibody interferes with the assay) with greater ADCC seen at 1 µg ml^−1^ than at higher antibody concentrations^26^ (Fig. 2c). To quantify antibody-dependent neutrophil phagocytosis (ADNP), SIV Env-RhmAb immune complex internalization was assessed by flow cytometry, with phagoscores indicating frequency of uptake^27^. The phagoscore for the four-RhmAb cocktail was the greatest (mean ± s.e.m. 56.55 ± 5.35), and ITS09.01-LS showed the highest phagoscore (44.40 ± 3.89) for an individual RhmAb (Fig. 2d). In general, cell binding ability correlated with ADCC, with the CD4bs-specific RhmAbs ranking highest; ADNP activity did not follow the same pattern, however, and these results provided rationale for inclusion of ITS09.01-LS in the in vivo study. Lower binding of the MPER-specific RhmAb ITS113.01-LS is likely due to limited accessibility of target epitopes on the cell surface^28^, but we rationalized its inclusion in the cocktail to be used in vivo due to its unique Env recognition site and SIV neutralizing ability^20^. Taken together, these results indicate that each SIV Env RhmAb has unique functional properties and that the combination of four RhmAbs with different SIV Env epitope targets binds to the surface of infected cells and mediates ADCC and ADNP at concentrations that are readily achievable in vivo.

### SIV Env RhmAb concentrations in vivo

**Extended Data Fig. 2:**
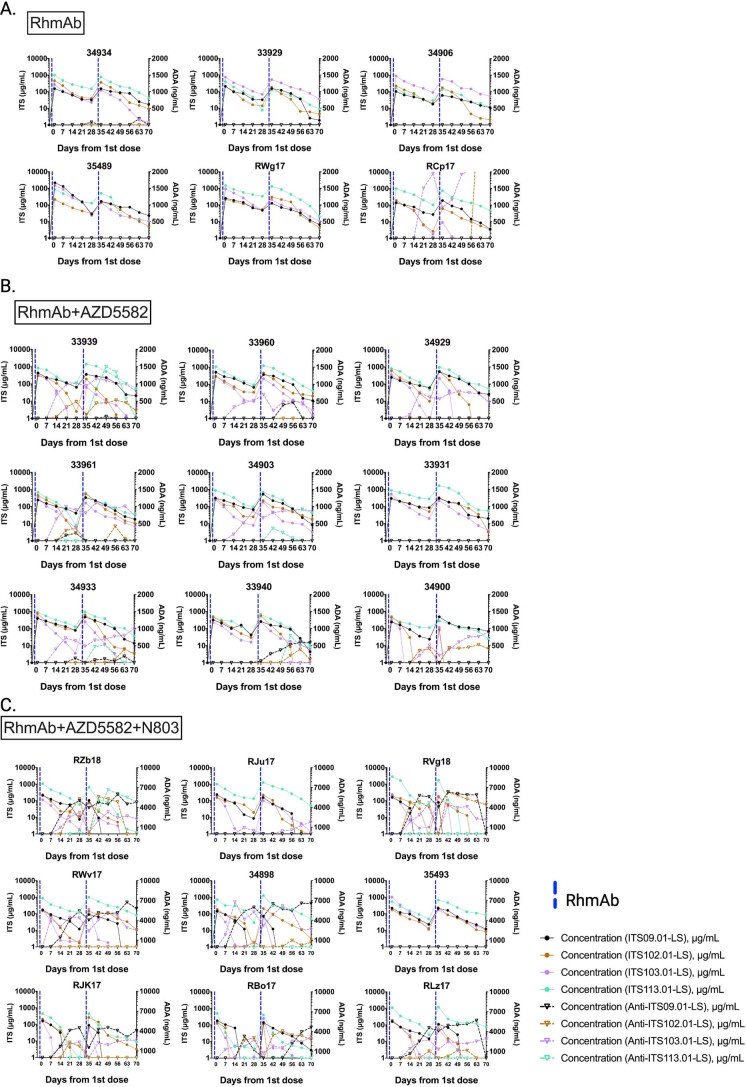
RhmAb pharmacokinetics and ADA measurements in individual macaques. Serum concentrations of RhmAbs and ADA measurements in individual RMs in RhmAb control group (**a**), RhmAb + AZD5582 group (**b**), RhmAb + AZD5582 + N-803 group (**c**). Solid lines = RhmAb concentration; dashed lines = ADA level. Vertical dashed lines represent the RhmAb administrations.

Serum concentrations of all four SIV Env RhmAbs peaked 24 h after s.c. injection and then declined before the second administration (Fig. 2e and Extended Data Fig. 2). Median C_max_, C_trough_ and half-life of each RhmAb in the three groups of RMs are shown in Table 2. Half-lives were mostly similar across groups, except for ITS103.01-LS with a median 3-d half-life in the RhmAb + AZD5582 + N-803 group compared to 7 d in the RhmAb control group (*P* = 0.0095) (Fig. 2f). The second injection of RhmAbs resulted in similar C_max_ and C_trough_ levels as the first within each group. Examination of the area under the curve (AUC) for concentrations of each of the RhmAbs over 70 d revealed lower antibody exposure in the RhmAb + AZD5582 + N-803 group compared to the RhmAb + AZD5582 group, with the exception of ITS113.01-LS that was similar (*P* = 0.0003 for ITS09.01-LS; *P* = 0.002 for ITS102.01-LS; *P* = 0.03 for ITS103.01-LS) (Fig. 2g).

**Table 2:** Pharmacokinetic Properties of SIV Env RhmAbs across Study Groups

	ITS09.01-LS			ITS102.01-LS			ITS103.01-LS			ITS113.01-LS		
Group	C_max_ (μg ml^−1^)	C_trough_ (μg ml^−1^)	Half-life (days)	C_max_ (μg ml^−1^)	C_trough_ (μg ml^−1^)	Half-life (days)	C_max_ (μg ml^−1^)	C_trough_ (μg ml^−1^)	Half-life (days)	C_max_ (μg ml^−1^)	C_trough_ (μg ml^−1^)	Half-life (days)
RhmAb	180	36	7.9	215	44	6.1	250	33	7.2	944	176	5.6
RhmAb + AZD5582	315	64	8.6	448	25	5.8	348	6	4.0	832	94	10.1
RhmAb + AZD5582 + N-803	170	8	7.8	184	1	5.8	179	1	3.1	877	49	5.7

**Extended Data Fig. 3:**
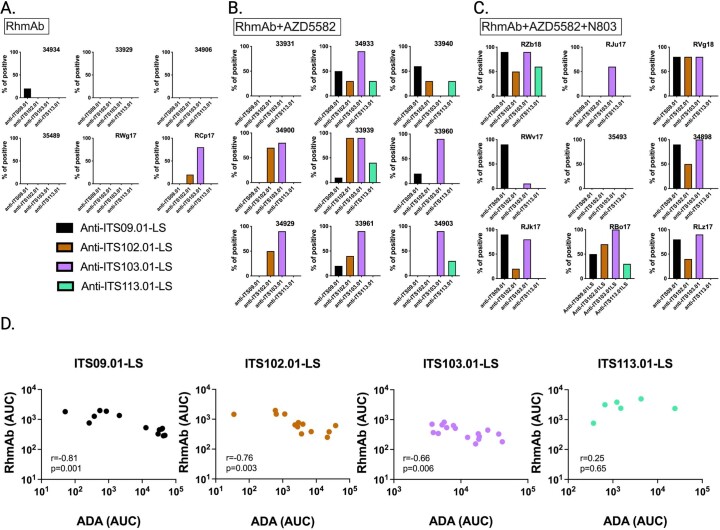
ADA measurements in individual macaques. **a–c**. ADA development in the three groups that received RhmAbs (% of time points where ADA was detected during the ten weeks of the intervention phase). **d**. Negative correlation between RhmAb serum concentrations (AUC) and ADA levels (AUC) (all groups combined) for all RhmAbs except ITS113.01-LS. Nonparametric two-sided Spearman correlation with 95% confidence interval was used for statistical analysis.

The development of anti-drug antibodies (ADAs) was monitored weekly for 10 weeks. Using anti-idiotype ELISAs, we determined that ADAs were infrequent in the RhmAb control group (2/6 RMs) but developed in eight of nine RMs in both the RhmAb + AZD5582 and RhmAb + AZD5582 + N-803 groups (Extended Data Figs. 2 and 3a–c). ITS103.01-LS elicited the most ADAs, and we found a positive correlation between the decline in serum RhmAb concentrations and ADA formation for ITS09.01-LS, ITS102.01-LS and ITS103.01-LS but not ITS113.01-LS (Extended Data Fig. 3d).

### Immunologic effects of RhmAb + AZD5582 ± N-803 treatment

**Extended Data Fig. 4:**
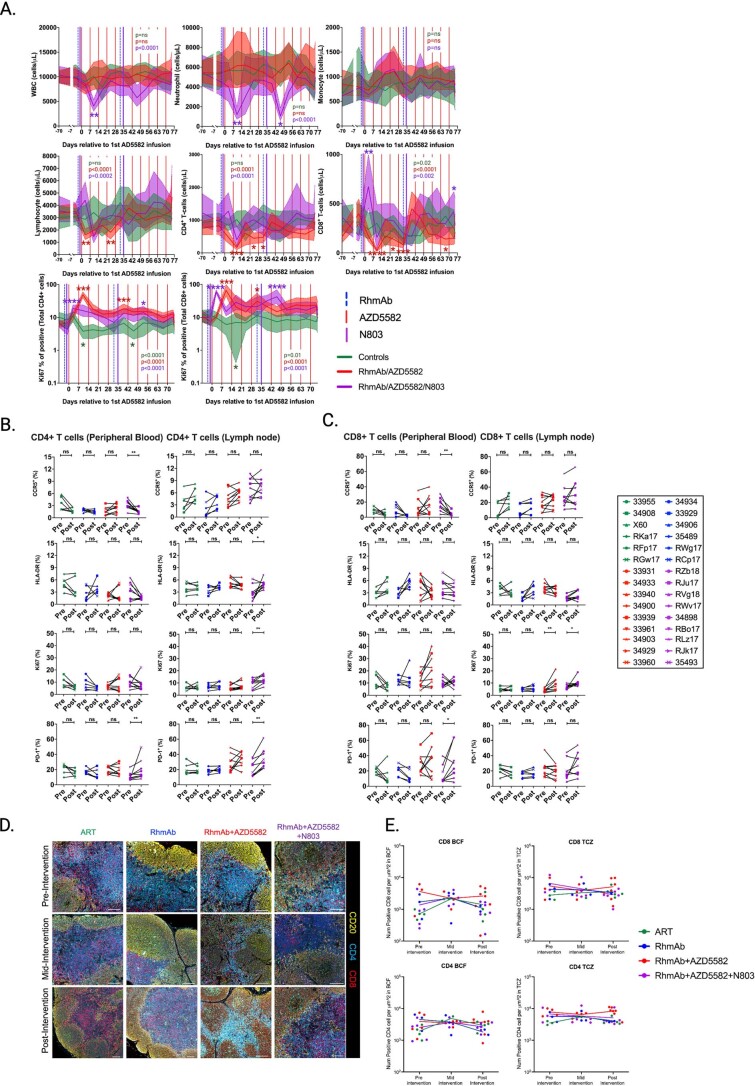
Longitudinal immune cell subsets and their phenotype. **a**. Longitudinal assessment of white blood cells (WBC), neutrophils, monocytes (top); lymphocytes, CD4^+^ T cells, and CD8^+^ T cells (middle); and Ki67 expression in CD4^+^ T cells (left), and CD8^+^ T cells (right) in peripheral blood. Line shows the mean with shading representing SEM. Statistical significance was determined using Friedman test with Dunn’s test for multiple comparisons. Friedman p value shown on the graphs with post-test comparisons shown by * p < 0.05; ** p < 0.01; *** p < 0.001; **** p < 0.0001. Controls (n = 12), RhmAb + AZD5582 (n = 9), and RhmAb + AZD5582 + N-803 (n = 9). Exact p values reported in Results, with the exception of i) Control group Ki67^+^CD4^+^ T cells: p = 0.004, day 10 vs −7 and p = 0.0004, day 46 vs −7; Ki67^+^CD8^+^ T cells: p = 0.009, day 10 vs −7; ii) RhmAb + AZD5582 group lymphocytes: p = 0.002, day 3 vs −5 and p = 0.007, day 24 vs −5; CD4^+^ T cells: p<0.0001, day 10 vs −5 and p = 0.03, days 24 and 32 vs −5; CD8^+^ T cells: p<0.0001, day 10 vs −5, p = 0.02, day 24 vs −5, p = 0.0002, day 32 vs −5, and p = 0.04, day 67 vs −5; Ki67^+^CD4^+^ T cells: p<0.0001, day 10 vs −7 and p<0.0001, day 39 vs −7; Ki67^+^CD8^+^ T cells: p<0.0001, day 10 vs −7 and p = 0.02, day 32 vs −7; iii) RhmAb + AZD5582 + N-803 group Ki67^+^CD4^+^ T cells: p = 0.001, day 3 vs −7 and p = 0.02, day 53 vs −7; Ki67^+^CD8^+^ T cells: p<0.0001, day 3 vs −7 and p < 0.0001, day 46 vs −7. **b**. Comparison of CCR5, HLA-DR, Ki67, and PD-1 expression pre- and post-intervention (weeks 78–87 and 101–105) in CD4^+^ T cells from peripheral blood (left) and LN (right). ART controls (n = 6), RhmAb controls (n = 6), RhmAb + AZD5582 (n = 9), and RhmAb + AZD5582 + N-803 (n = 9). Exact p values reported in Results, with the exception of blood CD4^+^CCR5^+^ and blood CD4^+^PD-1^+^ T cells for the RhmAb + AZD5582 + N-803 group (p = 0.004 and p = 0.008, respectively). **c**. Comparison of CCR5, HLA-DR, Ki67, and PD-1 expression pre- and post-intervention (weeks 78–87 and 101–105) in CD8^+^ T cells from peripheral blood (left) and LN (right). Exact p values reported in Results, with the exception of blood CD8^+^CCR5^+^ and blood CD8^+^PD-1^+^ T cells for the RhmAb + AZD5582 + N-803 group (p = 0.004 and p = 0.04, respectively). Statistical significance was determined using two-sided Wilcoxon matched-pairs signed-rank tests in (B) and (C). NS = non-significant. * p < 0.05; ** p < 0.01. **d**. Morphologic assessment of lymph nodes in each group. **e**. Levels of CD4^+^ and CD8^+^ T cells in lymph nodes comparing pre-, mid- and post-intervention time points in the RhmAb + AZD5582 and RhmAb + AZD5582 + N-803 groups and similar time points in ART and RhmAb controls. Pre-intervention: n = 3 (ART controls), n = 3 (RhmAb controls), n = 4 (RhmAb + AZD5582), n = 3 (RhmAb + AZD5582 + N-803). Mid-intervention: n = 2 (ART controls), n = 3 (RhmAb controls), n = 4 (RhmAb + AZD5582), n = 5 (RhmAb + AZD5582 + N-803). Post-intervention: n = 4 (ART controls), n = 2 (RhmAb controls), n = 7 (RhmAb + AZD5582), n = 6 (RhmAb + AZD5582 + N-803).

To investigate the immunological response to repeated RhmAb, RhmAb + AZD5582 and RhmAb +AZD5582 + N-803 administration, blood was collected throughout the intervention phase. Fine needle aspirations and biopsies of lymph nodes were also performed longitudinally to evaluate response to the treatments. Compared to baseline, RhmAb + AZD5582 + N-803 treatment resulted in significantly decreased white blood cell and neutrophil counts in circulation (*P* = 0.001 and *P* = 0.002, respectively, day 10 versus day −5, and *P* = 0.02 for neutrophils, day 46 versus day −5; Extended Data Fig. 4a). The RhmAb + AZD5582 + N-803 group also demonstrated a rapid increase in absolute CD8^+^ T cell count in blood (*P* = 0.003, day 3 versus day −5), with post-treatment levels higher than baseline (*P* = 0.04; Extended Data Fig. 4a). Treatment with RhmAb + AZD5582 in the absence of N-803 led to a decline in circulating lymphocytes, CD4^+^ T cells and CD8^+^ T cells, as indicated in Extended Data Fig. 4a. The frequency of proliferating CD4^+^ and CD8^+^ T cells, assessed through Ki-67 expression, increased in blood in both the RhmAb + AZD5582 + N-803 and RhmAb + AZD5582 groups (with selected statistical comparisons shown in Extended Data Fig. 4a). In contrast, in the control group, proliferating T cell levels were reduced or stable compared to baseline. Despite these changes, significant differences in Ki-67 expression in blood CD4^+^ and CD8^+^ T cells were not found comparing pre-treatment and post-treatment levels in any group (Extended Data Fig. 4b,c). In lymph nodes, however, Ki-67 expression was found to be significantly higher after treatment in CD4^+^ T cells in the group given N-803 (*P* = 0.008) and in CD8^+^ T cells from both treatment groups (*P* = 0.004 for RhmAb + AZD5582 and *P* = 0.04 for RhmAb + AZD5582 + N-803; Extended Data Fig. 4b,c). Lymph node CD4^+^ T cells from RMs treated with RhmAb + AZD5582 + N-803 also showed increases in other activation markers (HLA-DR, *P* = 0.01; PD-1, *P* = 0.004) compared to pre-treatment levels (Extended Data Fig. 4b). Similar changes were not observed after treatment in the RhmAb + AZD5582 or control groups. Histologic examination of lymph nodes before and after intervention showed preserved morphology in all groups (Extended Data Fig. 4d). Quantification of CD8α^+^ and CD4^+^ cells (Extended Data Fig. 4e) in the B cell follicle (BCF) and T cell zone (TCZ) revealed similar levels in all groups that remained stable over time.

### Functional markers in innate and adaptive cells

**Extended Data Fig. 5:**
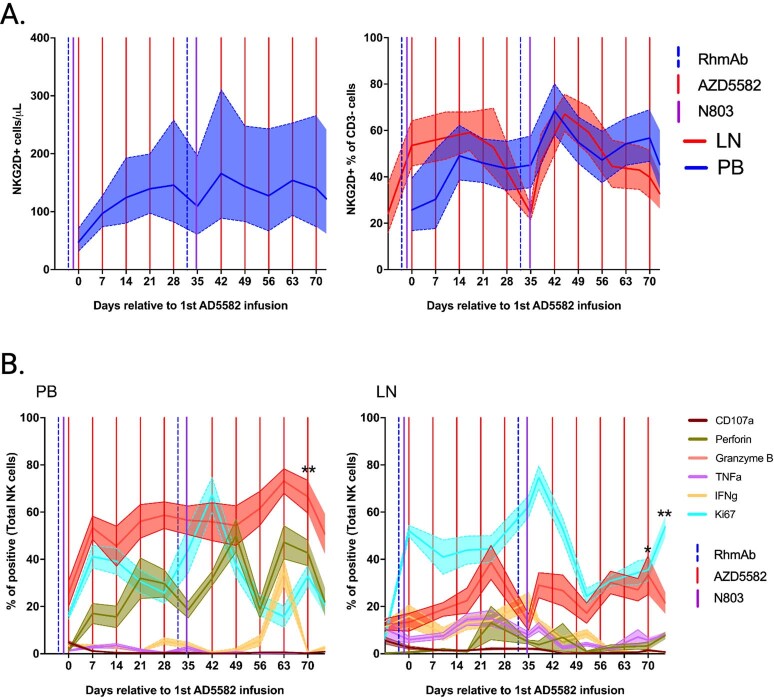
Innate effector cells. **a**. Longitudinal assessment of total NK cells in the blood of the RhmAb + AZD5582 + N-803 group (top left, n = 9). NK cell frequency in blood and lymph nodes from the RhmAb + AZD5582 + N-803 group (top right, n = 9). Line shows the mean with shading representing SEM. **b**. Longitudinal expression of functional markers in NK cells from peripheral blood (bottom left) and lymph nodes (bottom right) in the RhmAb + AZD5582 + N-803 group. Line shows the mean with shading representing SEM. Statistical significance was determined using two-sided Wilcoxon matched-pairs signed-rank test. * p < 0.05; ** p < 0.01.

NK cells and their expression of effector molecules were tracked longitudinally in animals receiving N-803. The frequency of NK cells in blood and lymph nodes increased after the first and second N-803 doses and then declined to baseline levels (Extended Data Fig. 5a). Ki-67 expression fluctuated in NK cells from both blood and lymph nodes and remained significantly elevated in lymph node NK cells at the end of the treatment period (*P* = 0.004, day 77 versus day 0; Extended Data Fig. 5b). The expression of granzyme B increased over time in circulating (*P* = 0.004, day 70 versus day 0) and lymph node (*P* = 0.01, day 70 versus day 0) NK cells, and NK cells from blood also showed increased levels of perforin at multiple timepoints (Extended Data Fig. 5b).

**Extended Data Fig. 6:**
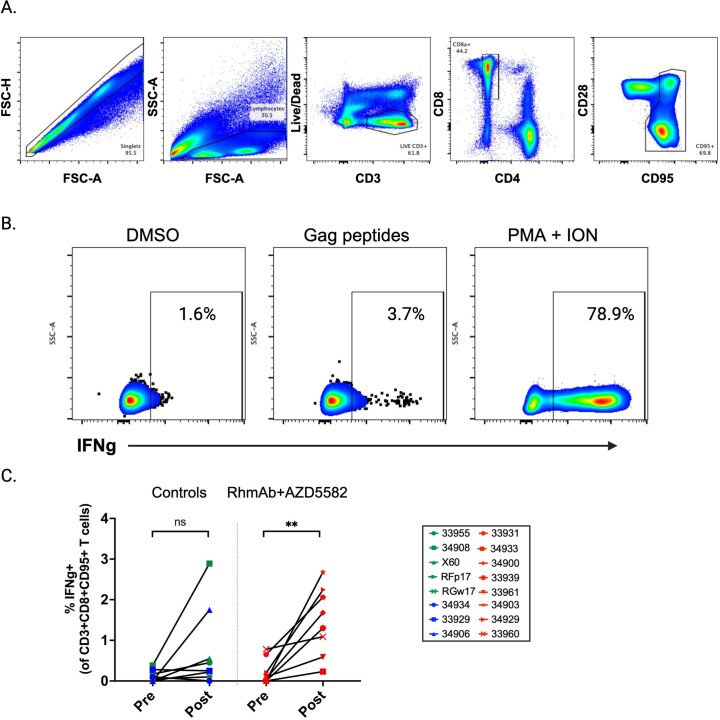
Adaptive effector cells. **a**. Gating strategy to identify CD8^+^CD95^+^ T cells. **b**. Representative gating for IFNg in CD8^+^CD95^+^ T cells following incubation with DMSO (negative control), SIV Gag peptides, and phorbal 12-myristate 13-acetate (PMA) + ionomycin (ION) (positive control). **c**. Frequencies of SIV-specific memory CD8^+^ T cells in the ART and RhmAb controls (n = 8, left) and RhmAb + AZD5582 group (n = 8, right). Data points shown are DMSO subtracted from the Gag condition at each time point and for each individual RM. Statistical significance was determined using the two-sided Wilcoxon matched-pairs signed-rank test. ** p < 0.01.

Intracellular cytokine staining was next performed to measure SIV-specific CD8^+^ T cell responses before and after intervention. Frequencies of memory CD95^+^CD8^+^ T cells expressing IFNγ, TNFα, CD107a or IL-2 upon stimulation with SIV Gag peptides were assessed (see Extended Data Fig. 6a,b for representative gating strategies). We did not find increases in expression of any of these markers in the ART and RhmAb control groups. However, the frequency of CD3^+^CD8^+^CD95^+^ T cells expressing IFNγ was significantly increased at the post-treatment timepoint in the RhmAb + AZD5582 group (Extended Data Fig. 6c). Only four macaques from the RhmAb + AZD5582 + N-803 group had sufficient cells for this assay, so no conclusions could be drawn for this limited dataset.

### Latency reversal during RhmAb + AZD5582 ± N-803 treatment

**Fig. 3:**
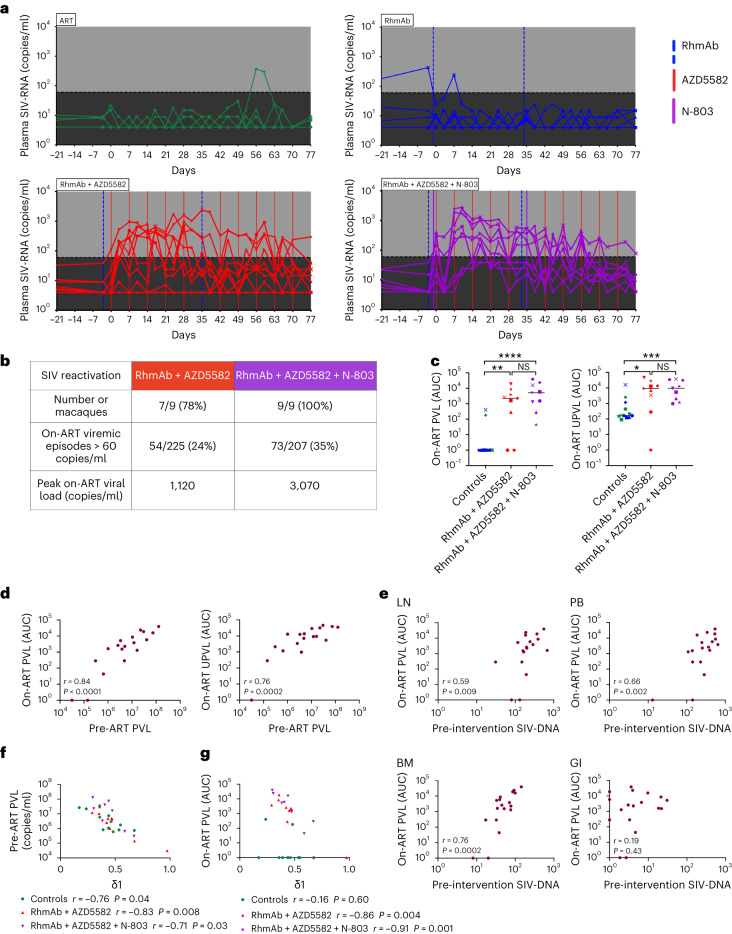
Latency reversal induced by AZD5582 and AZD5582 + N-803 in ART-suppressed SIV-infected macaques. **a**, Plasma SIV-RNA levels measured by the ultrasensitive viral load assay in ART-suppressed SIV-infected RMs during the intervention phase of the experiment. The black shading defines the difference between the LOD of conventional and ultrasensitive viral load assays (60 versus 4 copies per milliliter). Gray shading represents the period of ART administration. Blue vertical lines represent RhmAb administration; purple vertical lines represent N-803 administration; and red vertical lines represent AZD5582 infusions. The first N-803 dose was administered 3 d before AZD5582 and at the same time as RhmAbs. Green symbols: ART control group (*n* = 6); blue symbols: RhmAb control group (*n* = 6); red symbols: RhmAb + AZD5582 group (*n* = 9); purple symbols: RhmAb + AZD5582 + N-803 group (*n* = 9). **b**, Comparison of latency reversal in RhmAb + AZD5582 and RhmAb + AZD5582 + N-803 groups using ultrasensitive viral load assay results. **c**, Comparison of on-ART viremia (AUC) between groups during the intervention period (days 0–77). Both control groups (ART and RhmAbs) are combined. Results from both the standard PVL assay and the UPVL assay are shown. Controls (*n* = 12), RhmAb + AZD5582 (*n* = 9) and RhmAb + AZD5582 + N-803 (*n* = 9). Statistical significance was determined with a two-sided Mann–Whitney *U*-test. Horizontal lines represent the median. NS, non-significant. *P < 0.05; **P < 0.01; ***P < 0.001; ****P < 0.0001. **d**, Correlation between pre-ART (week 8) PVLs and on-ART viremia presented as AUC of plasma SIV-RNA levels measured by standard viral load assay (left) and ultrasensitive viral load assay (right) in all RMs that received AZD5582. **e**, Correlation between AUC of on-ART viremia during the intervention period in all RMs that received AZD5582 and CD4^+^ T-cell-associated SIV-DNA at weeks 81–87 (before AZD5582 or AZD5582 + N-803 administration) in lymph nodes (LNs), peripheral blood (PB), bone marrow (BM) and gastrointestinal tract (GI). **f**, Correlation between pre-ART (week 8) plasma SIV-RNA levels and *δ*_1_. **g**, Correlation between on-ART plasma viremia during intervention and *δ*_1_. Non-parametric two-sided Spearman correlation with 95% confidence interval was used for statistical analysis in **d**–**g**.

We next evaluated the latency reversal activity of AZD5582 in all treated RMs after 81–87 weeks of ART. Latency reversal was defined as a plasma SIV-RNA level of more than 60 copies per milliliter measured by standard viral load assay in the continued presence of ART (on-ART viremia) after a consistent period of viral suppression below this level. When AZD5582 was combined with SIV Env RhmAbs with or without N-803, we observed on-ART viremia in seven of nine (78%) and nine of nine (100%) RMs, respectively (Fig. 3a). The first episode of on-ART viremia occurred 72 h after the first dose of AZD5582 in the RhmAb + AZD5582 group. In four of nine RMs in the RhmAb + AZD5582 + N-803 group, on-ART viremia was observed before the first AZD5582 dose but after N-803 was administered (Fig. 3a). This finding suggests that N-803 itself may have reversed latency, although, without a group that received N-803 in the absence of AZD5582, the strength of this observation is limited. Previous studies do not support N-803 as a robust LRA in non-human primate (NHP) models^17,29^.

**Extended Data Fig. 7:**
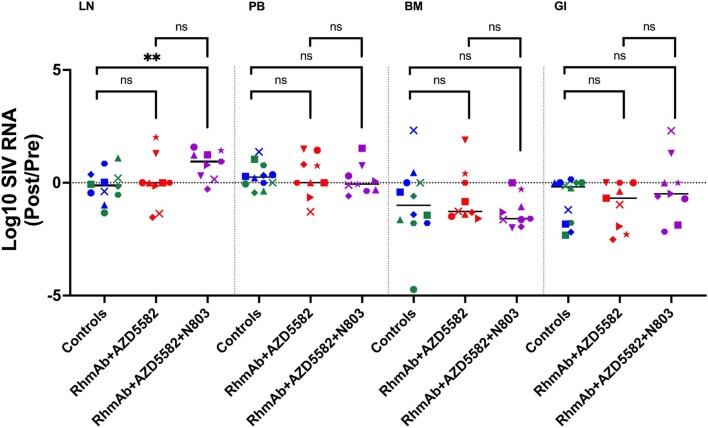
Cell-associated SIV-RNA assessment. Comparison of the fold change of SIV-RNA levels in CD4^+^ T cells isolated from LN, PB, BM, and GI tract between groups. Controls (n = 12), RhmAb + AZD5582 (n = 9), and RhmAb + AZD5582 + N-803 (n = 9). Statistical significance was determined with a two-sided Mann-Whitney U test. Horizontal lines represent the median. NS = non-significant. ** p < 0.01.

On-ART viremia was also measured by an ultrasensitive assay with a detection limit of 4 copies per milliliter. In the 6–8 weeks before AZD5582 ± N-803 treatment, 22 of 45 (49%) viral load measurements were less than 4 copies per milliliter, and 31 of 45 (69%) were less than 10 copies per milliliter, indicating strong viral suppression by ART. Of 225 viral loads measured during the intervention phase in the nine RhmAb + AZD5582-treated RMs, 54 (24%) were more than 60 copies per milliliter, with a maximum of 1,120 copies per milliliter. In the nine RhmAb + AZD5582 + N-803-treated RMs, 73 of 207 (35%) intervention phase viral load measurements were more than 60 copies per milliliter, with a maximum of 3,070 copies per milliliter (Fig. 3a,b). AUC of on-ART viremia (by both conventional and ultrasensitive viral load assays) demonstrated a significant increase in both groups that received AZD5582 compared to controls (*P* = 0.002 and *P* < 0.0001 for RhmAb + AZD5582 and RhmAb + AZD5582 + N-803 versus controls for PVL, respectively, and *P* = 0.02 and *P* = 0.0009 for RhmAb + AZD5582 and RhmAb + AZD5582 + N-803 versus controls for ultrasensitive plasma viral load (UPVL), respectively). Similar AUC levels were observed in the RhmAb + AZD5582 and RhmAb + AZD5582 + N-803 groups (Fig. 3c). To investigate latency reversal in tissues, we compared the fold change over the intervention period in SIV-RNA in CD4^+^ T cells isolated from peripheral blood, lymph nodes, bone marrow and gastrointestinal tract. RMs treated with RhmAb + AZD5582 + N-803 had a statistically significant fold increase in SIV-RNA in lymph node CD4^+^ T cells compared to controls (*P* = 0.007) (Extended Data Fig. 7). This finding is consistent with N-803 enhancing virus reactivation during AZD5582 treatment (demonstrated by on-ART viremic episodes and fraction of RMs with on-ART viremia) as well as the increase in CD4^+^ T cell activation in lymph nodes of RMs treated with N-803.

### Factors associated with magnitude of latency reversal

**Extended Data Fig. 8:**
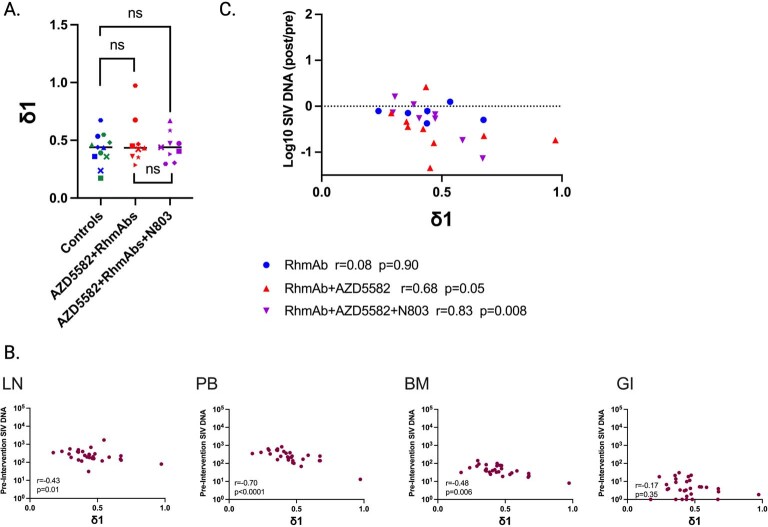
*δ*_1_ and reservoir levels pre-intervention. **a**. Comparison of ${\delta }_{1}$ between groups. Controls (n = 12), RhmAb + AZD5582 (n = 9), and RhmAb + AZD5582 + N-803 (n = 9). Statistical significance was determined with a two-sided Mann-Whitney U test. Horizontal lines represent the median. NS = non-significant. **b**. Correlation between ${\delta }_{1}$ and levels of CD4 + T cell-associated SIV-DNA in all RMs at weeks 81–87 before treatment with RhmAbs, AZD5582, or N-803 in lymph nodes (LN), peripheral blood (PB), bone marrow (BM), and gastrointestinal (GI) tract. **c**. Correlation between *δ*_1_ and the fold change in SIV-DNA in LN CD4^+^ T cells for each group that received RhmAbs. Nonparametric two-sided Spearman correlation with 95% confidence interval was used for statistical analysis in (B) and (C).

When both groups that received AZD5582 were considered together, a positive correlation was found between PVLs at the time of ART initiation and on-ART viremia AUC during AZD5582 treatment measured by conventional assay (*r* = 0.84, *P* < 0.0001) and ultrasensitive assay (*r* = 0.76, *P* = 0.0002) (Fig. 3d). As higher pre-ART viral loads may indicate increased reservoir seeding, we next evaluated the relationship between pre-intervention CD4^+^ T-cell-associated SIV-DNA levels and the magnitude of latency reversal and indeed found a positive correlation between on-ART viremia AUC and CD4^+^ T-cell-associated SIV-DNA in lymph nodes (*r* = 0.59, *P* = 0.009), peripheral blood (*r* = 0.66, *P* = 0.002) and bone marrow (*r* = 0.76, *P* = 0.0002) but not in gastrointestinal tract (*r* = 0.19, *P* = 0.43) (Fig. 3e). Viral loads during untreated HIV/SIV infection are dictated by the availability of target cells, which is, in turn, influenced by viral cytopathic effects, activation-induced cell death and killing of infected cells by immune effectors. The combined impact of these factors can be inferred by estimating the rate of the first-phase viral decay after ART initiation, ${\delta }_{1}$, which we also found to be inversely correlated with viral loads just before ART (Fig. 3f). Median *δ*_1_ was similar across groups (Extended Data Fig. 8a), with the range of values suggesting differential immune activation and effector responses across individual RMs. Notably, we found a negative correlation between ${\delta }_{1}$ and the AUC of on-ART viremia during the intervention phase in both treatment groups (RhmAb + AZD5582: *r* = −0.86, *P* = 0.004; RhmAb + AZD5582 + N-803: *r* = −0.91, *P* = 0.001) (Fig. 3g). These data suggest two non-exclusive hypotheses for the association of ${\delta }_{1}$ and magnitude of latency reversal during AZD5582 treatment: (1) a higher *δ*_1_ results in reduced reservoir seeding, which then influences the extent of latency reversal achievable by AZD5582; (2) a higher *δ*_1_ suggests more effective antiviral immune responses, which eliminate reactivated cells during AZD5582 treatment, resulting in reduced virus production. In support of the first hypothesis, we saw an inverse correlation between *δ*_1_ and pre-intervention CD4^+^ T-cell-associated SIV-DNA levels from blood, lymph nodes and bone marrow but not in the gastrointestinal tract (Extended Data Fig. 8b). We evaluated the second hypothesis through longitudinal measurement of infected cells before and after treatment with ART, RhmAbs and AZD5582 ± N-803, as described below.

### Impact on systemic SIV persistence

**Fig. 4:**
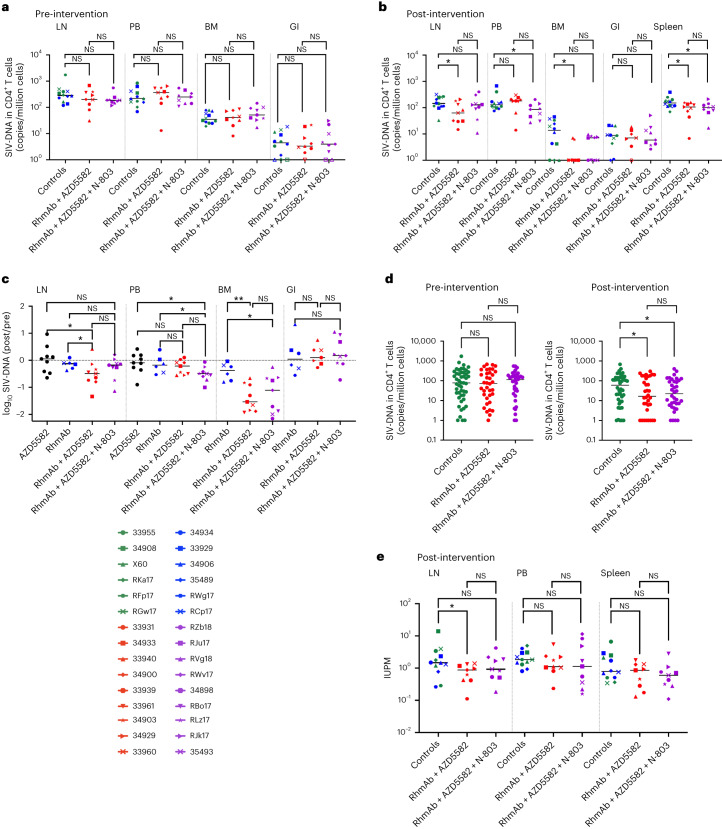
Viral reservoir assessment. **a**, Comparison of cell-associated SIV-DNA levels in CD4^+^ T cells isolated from lymph nodes (LNs), peripheral blood (PB), bone marrow (BM) and gastrointestinal tract (GI) between groups at weeks 81–87 (when all animals have received only ART—pre-intervention timepoint). Both control groups (ART and RhmAbs) are combined. Controls (*n* = 12), RhmAb + AZD5582 (*n* = 9) and RhmAb + AZD5582 + N-803 (*n* = 9). **b**, Comparison of cell-associated SIV-DNA levels in CD4^+^ T cells isolated from LN, PB, BM, GI tract and spleen at weeks 101–105 (after all interventions have been administered—post-intervention timepoint). Controls (*n* = 12), RhmAb + AZD5582 (*n* = 9) and RhmAb + AZD5582 + N-803 (*n* = 9). **c**, Comparison of the fold change of SIV-DNA levels in CD4^+^ T cells isolated from LN, PB, BM and GI tract between groups that received RhmAbs. For LN and PB, data from a historical group of RMs given AZD5582 are shown only for comparison. AZD5582 (*n* = 9), RhmAb controls (*n* = 6), RhmAb + AZD5582 (*n* = 9), RhmAb + AZD5582 + N-803 (*n* = 9). **d**, Comparison of cell-associated SIV-DNA levels in CD4^+^ T cells isolated from PB + LN + BM + GI (combined, to approximate the total body SIV-DNA pool) before intervention (left) and after intervention (right). Controls (*n* = 12), RhmAb + AZD5582 (*n* = 9), RhmAb + AZD5582 + N-803 (*n* = 9). **e**, Frequencies of CD4^+^ T cells from LN, PB and spleen with replication-competent SIV determined by quantitative viral outgrowth assay at weeks 101–105 (after all interventions have been administered—post-intervention timepoint). Controls (*n* = 12), RhmAb + AZD5582 (*n* = 9), RhmAb + AZD5582 + N-803 (*n* = 9). Statistical significance was determined using the Mann–Whitney *U*-test in **a**–**c** and **e** and two-way ANOVA with Tukey’s multiple comparisons test in **d**. Horizontal lines represent the median. NS, non-significant; IUPM, Infectious Units Per Million CD4^+^ T cells. *P < 0.05; **P < 0.01.

Given the high levels of SIV Env RhmAbs achieved in circulation and the evidence for substantial latency reversal in RMs receiving AZD5582 with or without N-803, we next assessed the impact of these therapies on SIV persistence. The ART control and RhmAb control groups were combined for these analyses to increase power as (expectedly) neither group demonstrated evidence of latency reversal, and statistically significant differences in any reservoir measurements between these two groups were not observed. We measured SIV-DNA in CD4^+^ T cells isolated from lymph nodes, peripheral blood, bone marrow and gastrointestinal tract before and after the intervention. We also quantified SIV-DNA in CD4^+^ T cells from spleen after treatment. There were no significant differences between groups in each anatomic site at baseline (Fig. 4a). In contrast, levels of CD4^+^ T-cell-associated SIV-DNA in lymph nodes (*P* = 0.02), spleen (*P* = 0.04) and bone marrow (*P* = 0.01) were significantly lower in RMs that received RhmAb + AZD5582 compared to controls after intervention (Fig. 4b). RMs that received N-803 in addition to RhmAbs and AZD5582 similarly had lower post-intervention SIV-DNA in CD4^+^ T cells from spleen (*P* = 0.04) and also peripheral blood (*P* = 0.04) but not lymph nodes (*P* = 0.42) in comparison to controls. Dichotomous results were seen in bone marrow, with SIV-DNA levels similar to controls in five RMs in the RhmAb + AZD5582 + N-803 group and undetectable SIV-DNA levels in the four remaining RMs (similar to the RhmAb + AZD5582 group). SIV-DNA measurements in CD4^+^ T cells from the gastrointestinal tract did not differ post intervention.

We next calculated the fold change over the intervention period in SIV-DNA in CD4^+^ T cells in RMs that received RhmAb + AZD5582 or RhmAb + AZD5582 + N-803 compared to RMs that received RhmAbs alone as well as a historical group of RMs that received AZD5582 only at the same dose and interval^16^ to compare combination versus single interventions. We found a significant fold reduction in the SIV-DNA reservoir size in lymph nodes in the RhmAb + AZD5582 group versus both the RhmAb control and AZD5582-only groups (*P* = 0.04 for each comparison) (Fig. 4c). A reduction in the SIV-DNA reservoir size in blood was seen in the RhmAb + AZD5582 + N-803 group compared to both the RhmAb control and AZD5582-only groups (*P* = 0.05 and *P* = 0.02, respectively) (Fig. 4c). In both the RhmAb + AZD5582 and RhmAb + AZD5582 + N-803 groups, we also noted a greater fold change in bone marrow CD4^+^ T-cell-associated SIV-DNA compared to RhmAb controls (*P* = 0.002 and *P* = 0.04, respectively) (Fig. 4c). No differences were seen in the gastrointestinal tract, consistent with the results shown in Fig. 4b. These data suggest that the combination therapies were more effective in clearing infected cells than either RhmAbs or AZD5582 alone and that AZD5582 + N-803 may better target the reservoir in blood, whereas, somewhat unexpectedly, the impact of AZD5582 in the absence of N-803 was directed to lymph nodes. The rate of first-phase viral decay after ART initiation, *δ*_1_, positively correlated with the fold change in lymph node CD4^+^ T cell SIV-DNA in both the RhmAb + AZD5582 (*r* = 0.68, *P* = 0.05) and RhmAb + AZD5582 + N-803 groups (*r* = 0.83, *P* = 0.008) but not in RhmAb controls (*r* = 0.08, *P* = 0.9) (Extended Data Fig. 8c). This suggests to us that factors contributing to viral decay on ART, including cytolytic immune responses, may influence the extent of infected cell elimination in addition to the magnitude of latency reversal in RMs that received AZD5582 ± N-803 combined with RhmAbs. The changes in NK and CD8^+^ T cell effector functions over the course of treatment (shown in Extended Data Figs. 5 and 6) may also have contributed to clearance of SIV-infected CD4^+^ T cells.

To approximate the total body infected cell pool, we graphed the sum of all SIV-DNA results from blood, lymph nodes, bone marrow and gastrointestinal tract both before and after intervention, recognizing as a limitation that this sum does not account for the proportion of CD4^+^ T cells at each anatomic site. Groups did not differ before intervention (when all RMs had only received ART); however, the estimated total body SIV-DNA level was reduced after administration of either RhmAb + AZD5582 or RhmAb + AZD5582 + N-803 compared to controls (*P* = 0.03 and *P* = 0.02, respectively) (Fig. 4d). Splenic CD4^+^ T cells were not included in this analysis, as pre-intervention sampling was not performed.

As polymerase chain reaction (PCR) for SIV*gag* DNA quantifies both replication-competent and replication-defective viruses, we next performed virus outgrowth assays using CD4^+^ T cells from lymph nodes, peripheral blood and spleen. Based on the cell number requirements for the quantitative virus outgrowth assay (QVOA), this approach could be used only at necropsy. Compared to controls, the frequency of replication-competent SIV in lymph node CD4^+^ T cells was reduced in the RhmAb + AZD5582 group (*P* = 0.04) (Fig. 4e). Further differences between treatment groups and controls were not observed, however, suggesting that clearance of cells carrying replication-competent virus was not achieved across multiple anatomic sites.

## Discussion

The key obstacle to HIV cure is the presence of a persistent reservoir of latently infected CD4^+^ T cells that harbor integrated, replication-competent provirus, which induce a rapid rebound of virus replication upon ART interruption. The current study demonstrates, as proof of concept, that a reduction of the SIV reservoir in lymph nodes after long-term ART is achievable with a combination of AZD5582 and four SIV Env-specific RhmAbs. Further enhancement of this approach could provide a safe and effective means to clear infected CD4^+^ T cells in tissues of people living with HIV. Despite the large number of research studies focused on LRAs, none has shown meaningful reductions in persistent virus^4^^,5,7,8–13,15^^,17^^,18^^,30^, indicating that the viral cytopathic effect and/or endogenous immune response stimulated by latency reversal is insufficient to eliminate cells with induced viral expression. In the current study, we took advantage of second-generation rhesus-derived mAbs with non-overlapping binding sites on the Env trimer and demonstrated their capacity to bind to SIV-infected cells and mediate both ADCC and ADNP. The high levels and durability of RhmAbs in circulation after s.c. injection of macaques were similar to those found in humans who received i.v. infusions of broadly neutralizing antibodies (bNAbs)^31^. We were initially uncertain how the RhmAb cocktail would impact the detection of on-ART viremia during AZD5582 treatment, but we did not find a substantial difference compared to AZD5582 alone^16^. The magnitude of latency reversal was directly associated with pre-ART viral loads and SIV-DNA containing CD4^+^ T cells after approximately 18 months of ART and negatively correlated with the rate of first-phase viral decline upon ART initiation, ${\delta }_{1}$. Although the magnitude of on-ART viremia in each animal was not correlated with reservoir size after intervention, fold change of SIV-DNA in lymph node CD4^+^ T cells positively correlated with ${\delta }_{1}$, suggesting that animal-specific immune responses influence the elimination of infected cells in addition to the degree of latency reversal. Increases in cytolytic molecules in CD8^+^ T cells during treatment with AZD5582 and in NK cells during treatment with AZD5582 + N-803 could also be important in reducing the SIV reservoir.

Combinations of LRAs that lead to more robust virus reactivation have been studied ex vivo and in vivo^30,32^. We have shown that treating ART-suppressed, SIV-infected macaques with AZD5582 and the anti-CD8α monoclonal antibody MT07R1 (that depletes CD8^+^ T and NK cells) results in increased on-ART viremic events compared to AZD5582 alone^33^ and, furthermore, that a powerful latency reversal ability of N-803 is revealed through CD8α cell depletion^17^. In the current study, we hypothesized that combining AZD5582 and N-803 to activate the ncNF-κB pathway and the multiple signaling pathways downstream of IL-15R engagement would enhance latency reversal and promote activation/proliferation of NK cells to support the functional activity of SIV RhmAbs in clearing infected CD4^+^ T cells (for example, through ADCC). We found that adding N-803 to AZD5582 led to latency reversal in 100% of treated animals compared to 78% treated with AZD5582 alone as well as a significant fold increase in SIV-RNA in lymph node CD4^+^ T cells compared to controls. Despite the increased frequency of NK cells in lymph nodes, including NK cells with cytotoxic effector molecules (e.g., granzyme B), after treatment with N-803, we did not find a change in infected cell pool size in lymph nodes in the RhmAb + AZD5582 + N-803 group. SIV-DNA in peripheral blood CD4^+^ T cells was lower in RMs given N-803 versus controls, and a greater fold reduction was also observed in this compartment; however, this change was not reflected in the virus outgrowth assay that quantifies replication-competent virus. It is possible that the proliferative effect of N-803 on CD4^+^ T cells resulted in clonal expansion of latently infected cells and mitigated the modest reservoir clearance effect of RhmAb + AZD5582 treatment. It is also possible that cells containing defective virus were preferentially induced to express SIV Env^34^ and eliminated by SIV RhmAbs, whereas cells containing inducible replication-competent SIV remained quiescent. Finally, we posit that the immune activation induced by N-803 in combination with AZD5582 contributed to the higher levels of ADA and, consequently, lower exposure to ITS09.01-LS, ITS102.01-LS and ITS103.01-LS in the RhmAb + AZD5582 + N-803 group, potentially reducing the interaction of these RhmAbs with reactivated cells.

In a small number of animals, a few viral load blips after ART suppression, but before the intervention phase, were observed, and two macaques that did not receive an LRA showed transient on-ART viremia (one each in the ART control and RhmAb control groups). These observations might indicate a lower reactivation threshold or less deep state of latency in this experiment, despite the long period of ART treatment, permitting increased activity of both AZD5582 and N-803. This conjecture would be supported by our data from SHIV.C.CH505 infection, with rapid and durable suppression of viremia on ART and also no episodes of on-ART viremia during AZD5582 treatment^18^. In close to half of RMs in the RhmAb + AZD5582 + N-803 group, on-ART viremia was seen after the first N-803 dose but before AZD5582 was given, possibly reflecting a differential susceptibility to virus reactivation compared to previous research^17^. We note that the dose of N-803 used here and in other NHP studies^17,29^ is higher than what has been tested in clinical trials enrolling people with HIV^35^, which may partially account for the absence of on-ART viremia (>60 copies per milliliter) in N-803-treated humans, and leaving open the question of N-803-driven clonal expansion of lymph node CD4^+^ T cells at the clinical dose used.

Previous research showed that the combination of a TLR7 agonist and bNAb reduces viral DNA in lymph nodes of ART-suppressed SHIV-infected macaques compared to controls, with ART started 1 week after infection^36^. Differently from that study, however, we show that the RhmAb + AZD5582 combination reduced lymph node reservoirs in the setting of highly pathogenic SIV and ART initiation at 8 weeks after infection. Interestingly, when ART initiation was delayed for 1 year after SHIV infection, the TLR7 agonist + bNAb approach did not lower viral DNA in peripheral blood mononuclear cells (PBMCs), colorectal tissue or lymph nodes compared to pre-intervention levels^37^. Notably, median pre-ART viremia (a proxy of reservoir size after ART) in this SHIV chronic infection model was 3 logs lower than in the current study, highlighting the very high bar that we set to assess an impact on tissue reservoirs by using SIV and ART started in early chronic infection. Given this high bar, the relatively modest reduction in replication-competent SIV that we observed is not surprising. Future research into the potential of longer-term administration and/or repeated cycles of these agents to promote additional infected cell clearance and delay viral rebound would be informative.

In both intervention groups, the largest reduction in infected cells (by SIV-DNA) was found in the bone marrow, and, conversely, infected CD4^+^ T cells in the gastrointestinal tract appeared to be unaffected. These results raise the possibility that heterogeneous distribution of SIV RhmAbs in tissues and/or tissue-specific responses to the LRAs used contribute to the observed effects on SIV reservoir. It is tempting to speculate that the SIV RhmAbs combined with AZD5582 produced a ‘vaccinal effect’ that led to the increased frequency of SIV-specific memory CD8^+^IFNγ^+^ T cells and subsequent infected CD4^+^ T cell clearance. The two macaques in the RhmAb + AZD5582 group (33931 and 33939) with the lowest pre-ART viral loads and among the lowest levels of infected CD4^+^ T cells across anatomic sites before intervention were the only ones in the group that did not exhibit on-ART viremia during AZD5582 treatment but still showed a decline in SIV-DNA in lymph nodes and bone marrow similar to the rest of their group. Low-level viremia below 60 copies per milliliter was observed in one of these animals but not the other. Both had evidence of increased SIV-specific CD8^+^ T cells at the end of the study, suggesting that antigen expression may occur during AZD5582 treatment without high-level viremia.

A limitation of this study is that we did not incorporate ART interruption into our experimental design and, therefore, cannot report the impact of our approach on viral rebound dynamics. Without these results, determining whether discordant results obtained from different viral assays (SIV*gag* DNA PCR and QVOA) after intervention reflect true biologic differences versus assay sensitivity and/or dynamic range is challenging. Furthermore, although we attempted to evaluate replication-competent reservoirs in multiple tissues at study end, sufficient numbers of CD4^+^ T cells could not be reliably isolated from the gastrointestinal tract or bone marrow to perform quantitative outgrowth assays. We also did not obtain brain tissue and did not include cells of the myeloid lineage in our investigations.

AZD5582 is now a well-validated tool to study the effects of latency reversal in vivo in combination with immunotherapies or interventions designed to clear persistently infected, reactivated cells. In addition, efforts are currently underway to bring an IAPi to phase 1 testing and allow translation of these advances to human clinical trials. With the exception of stem cell transplant used for oncologic indications, no treatment has been shown to eliminate the replication-competent HIV reservoir. Hope for an HIV cure has come from studying elite controllers and other non-progressors as well as from experiments in relevant animal models. The results that we describe using pathogenic SIV infection and late ART initiation provide novel proof of concept for the potential of the latency reversal and clearance HIV cure strategy. Next steps for this approach include NHP studies testing novel LRA combinations, repeated cycles of LRA treatment, vectored delivery of mAbs, strategies to reduce ADAs, more effective killing and immune augmentation agents and earlier intervention to prevent reservoir establishment. These preclinical studies, along with parallel safety testing of IAPi in human clinical trials, may aid in the progress toward an accessible cure for HIV.

## Methods

### Experimental design

This study aimed to reverse latency using the IAPi/SMACm AZD5582 as an HIV/SIV latency-reversing agent in vivo and clear or eliminate latently infected AZD5582-reversed CD4^+^ T cells. To this end, 30 RMs (*Macaca mulatta*) were used. Animals were infected with SIV, and viremia was durably suppressed by ART. AZD5582 with or without the IL-15 superagonist N-803 was administered to infected, ART-suppressed animals that were then assayed for changes associated with reactivation of the viral reservoir. RMs infected with SIV were housed at the Emory National Primate Research Center and treated according to Emory University and Emory National Primate Research Center regulations. Animal care facilities are accredited by the US Department of Agriculture and the Association for Assessment and Accreditation of Laboratory Animal Care International. The Emory University Institutional Animal Care and Use Committee approved this study (PROTO201700286).

### SIV infection of RMs

Thirty male and female (25 males and five females) Indian RMs, 2–5 years of age, with the exclusion of Mamu B*08-positive and Mamu B*17-positive animals, were enrolled in this study (Fig. 1c). RMs were i.v. infected with 3 × 10^3^ TCID_50_ of SIV_mac239_, which was kindly provided by Koen Van Rompay. SIV_mac239_ stock was titrated in vitro for viral infectivity by standard endpoint titration on CEMx174 cells. The TCID_50_ was calculated by the method of Reed and Meunch^38^. Standard SIV_mac239_ plasma viral load quantification was performed regularly throughout the study and three times per week during the AZD5582 treatment period in the Translational Virology Core Laboratory of the Emory Center for AIDS Research using a standard quantitative real-time PCR (qPCR) assay (LOD: 60 copies per millilter of plasma). Ultrasensitive SIV_mac239_ plasma viral load quantification (LOD: 4 copies per milliliter of plasma^39^) was performed for 2–3 timepoints before AZD5582 treatment longitudinally during AZD5582 treatment.

### ART, RhmAbs, AZD5582 and N-803 treatment

RMs were treated with a potent three-drug ART regimen initiated 56 d after infection, consisting of two reverse transcriptase inhibitors, tenofovir disoproxil fumarate (20 mg kg^−1^) and emtricitabine (40 mg kg^−1^), plus the integrase inhibitor dolutegravir (2.5 mg kg^−1^). ART was administered once daily via the s.c. route. Nine RMs were treated with a cocktail of four SIV Env-targeting RhmAbs and AZD5582 (RhmAb + AZD5582 group); nine RMs were treated with RhmAbs and AZD5582 + N-803 (RhmAb + AZD5582 + N-803 group); six RMs were treated with RhmAbs without LRAs (RhmAb controls); and six RMs did not receive any treatment except ART (ART controls). AZD5582 was infused weekly at 0.1 mg kg^−1^ i.v. for five consecutive weeks. After 1 week of rest, this cycle was repeated once. RhmAbs were given at 20 mg kg^−1^ s.c. each (total of 80 mg kg^−1^) before the first five doses of AZD5582 and before the second five doses of AZD5582. N-803 was administered at 100 μg kg^−1^ s.c. at the same time as RhmAbs. The first dose of N-803 was given 3 d before the first AZD5582 infusion, and one RM displayed generalized ecchymosis in the setting of reduced platelets and increased creatinine 2 d after AZD5582. Milder ecchymosis and/or petechiae were observed in two additional animals in the same timeframe. The second dose of N-803 was, therefore, given 1 week before the sixth AZD5582 infusion to avoid potential interactions, and no adverse events were observed during the second cycle of treatments. All animals were killed 1–2 weeks after the last treatment.

### Sample collection and processing

EDTA-anticoagulated blood samples were collected regularly and used for a complete blood count, routine chemical analysis and immunostaining, with plasma separated by centrifugation within 1 h of phlebotomy. At the end of the studies, tissue samples were collected from lymph nodes, spleen, bone marrow and gastrointestinal tract. After two washes in RPMI and removal of connective and fat tissues, lymph nodes were grinded using a 70-μm cell strainer. PBMCs and bone marrow mononuclear cells were prepared by density gradient centrifugation. Gastrointestinal tract specimens were cleaned and enzymatically digested with mononuclear cells and then prepared by density gradient centrifugation^40^. CD4^+^ T cells were negatively selected from fresh or frozen cell suspension using magnetically labeled microbeads and subsequent column purification according to the manufacturer’s protocol (Miltenyi Biotec).

### SIV infection of A66 cells

The infection of A66 cells was performed as previously described^25^. In brief, SIV_mac239_ virus stocks grown in rhesus PBMCs were titrated to determine the input required for optimal viral gene expression within 72 h after infection of A66 cells as measured by intracellular p27 expression (WNPRC Immunology Services). A66 cells were incubated with 33 ng ml^−1^ of p27 for 4 h at 37 °C and 5% CO_2_ in the presence of DEAE-Dextran (10 μg ml^−1^, Sigma-Aldrich). The cells were subsequently resuspended at 0.33 × 10^6^ per milliliter and cultured for 3 d in complete medium containing 10 μg ml^−1^ DEAE-Dextran. Infection was monitored by measuring the frequency of cells expressing intracellular p27, and the assay was considered reliable if the percentage of viable p27^+^ target cells was ≥10%. Assay data generated using infected cells were normalized to the frequency of live target cells positive for intracellular p27.

### ICABA

ICABA was used to evaluate the ability of RhmAbs to bind Env on the surface of SIV_mac239_-infected cells^25^. A66 cells incubated in the absence of virus (mock-infected) were used as a negative control. Infected and mock-infected cells were washed in PBS, dispensed into 96-well V-bottom plates at 2 × 10^5^ cells per well and incubated with 10 μg ml^−1^ or 1 μg ml^−1^ of indicated RhmAbs for 2 h at 37 °C. The cocktail of four RhmAbs was tested at 40 μg ml^−1^ or 4 µg ml^−1^ of total IgG (10 μg ml^−1^ or 1 µg ml^−1^ of each RhmAb, respectively). After two washes with 250 μl per well, cells were stained with vital dye (Live/Dead Fixable Aqua Dead Cell Stain, Invitrogen) to exclude non-viable cells from subsequent analysis. Cells were permeabilized using Cytofix/Cytoperm (BD Biosciences) and anti-p27-FITC antibody (WNPRC Immunology Services, 1:500 dilution in 1× Cytoperm Solution, BD Biosciences), and secondary phycoerythrin (PE)-conjugated antibody (goat anti-human Ig Fc-PE, eBioscience, final dilution 1:400) were added to each well and incubated in the dark for 25 min at 4 °C. Cells were washed three times with Cytoperm wash solution and resuspended in PBS/1% paraformaldehyde. Samples were acquired within 24 h using a BD Fortessa cytometer. A minimum of 50,000 total events was acquired. Gates were set to include singlet and live events. Data analysis was performed using FlowJo 9.6.6 software (BD Biosciences). Final data represents the PE mean fluorescence intensity (MFI) of binding of IgG RhmAbs to SIV Env, after normalization by subtraction of the PE MFI observed for cells stained with the secondary antibody alone. Assays were repeated twice. The negative control anti-desipramine (DSP) mAb was used at 10 µg ml^−1^ and 1 µg ml^−1^ or 40 µg ml^−1^ and 4 µg ml^−1^.

### ICE ADCC assay

An ICE assay was used to measure ADCC activity of RhmAb as previously described^25^. SIV_mac239_-infected or mock-infected A66 cells were used as targets, and the NK92RhCD16 cell line rested overnight in media supplemented with IL-2 (at a final concentration of 100 U ml^−1^) (PeproTech) was used as effector cells. Infected and uninfected target cells were washed in R10 and labeled with a fluorescent target cell marker (TFL4, OncoImmunin) and a viability marker (NFL1, OncoImmunin) for 15 min at 37 °C, as specified by the manufacturer. Cells were washed in R10 and adjusted to a concentration of 0.2 × 10^6^ cells per milliliter. NK92RhCD16 cells were then added to target cells at an effector:target ratio of 10:1 (2 × 10^6^ cells per milliliter). The cell suspension was plated in V-bottom 96-well plates and co-cultured with each RhmAb at a starting concentration of 100 μg ml^−1^ with six subsequent dilutions at 1:5. The cocktail of four RhmAbs was tested starting with 80 µg ml^−1^ of total IgG (20 µg ml^−1^ of each RhmAb). Co-cultures were incubated for 6 h at 37 °C in 5% CO_2_. After the incubation period, cells were washed and resuspended in 100 μl per well Cytofix/Cytoperm (BD Biosciences), incubated in the dark for 20 min at 4 °C, washed in 1× Cytoperm wash solution (BD Biosciences) and co-incubated with anti-p27 antibody (WNPRC Immunology Services) to a final dilution of 1:500 and then incubated in the dark for 25 min at 4 °C. Three washes were performed with Cytoperm wash solution before resuspending the cells in 125 μl of PBS–1% paraformaldehyde for acquisition. Samples were acquired within 24 h using a BD Fortessa cytometer. Data analysis was performed using FlowJo 9.6.6 software. Mock-infected cells were used to appropriately position live p27^+^ cell gates. Specific killing (%ADCC ICE) was determined by the reduction in percentage of p27^+^ cells in the presence of RhmAbs after controlling for non-specific killing according to the following formula: percent specific killing = [(frequency of p27^+^ cells in wells containing targets and effectors alone − frequency of p27^+^ cells in wells containing targets and effectors plus RhmAbs) / frequency of p27^+^ cells in wells containing targets and effectors alone] × 100. Anti-DSP mAb starting at 80 µg ml^−1^ was used as a negative control.

### Antibody-dependent neutrophil phagocytosis

Biotinylated gp140 was incubated with 1 μm of fluorescent neutravidin beads (Invitrogen, F8776) for 4 h at 4 °C. Beads were subsequently spun down and washed twice in PBS–BSA to remove excess unbound antigen. Beads were then divided equally into five portions. Each of the four RhmAbs was added to beads and incubated for 2 h at 37 °C. The final portion was used as an antibody negative control. Beads were spun down and washed twice in PBS–BSA to remove excess unbound RhmAbs and then resuspended at a final dilution of 1:100 in PBS–BSA. Then, 5 × 10^5^ red blood cell (RBC)-depleted blood cells were added to bead-coated RhmAbs and incubated for 4 h in a final volume of 200 μl. Cells were washed and stained with CD66abcd and fixed with 100 μl of 4% paraformaldehyde before plates were analyzed by flow cytometry on a FACSymphony A5. At least 200,000 cells were acquired for data analysis. A phagocytic score was determined by gating on events representing cells and calculated as follows: % bead-positive neutrophils ${\rm{\times }}$ geometric MFI (gMFI) of bead-positive neutrophils. For ease of presentation, these scores were then divided by 10^5^.

### RhmAbs pharmacokinetics in NHPs

The total concentration of the RhmAbs in serum was measured by a sandwich immunoassay using anti-RhmAb (mouse) mAbs immobilized for capture and anti-rhesus-horseradish peroxidase (HRP) for detection^19^. The lower limit of quantification was 3.0 ng ml^−1^.

### ADA assay

ADAs to individual RhmAbs were measured by a sandwich immunoassay using RhmAb (rhesus) Fab immobilized mAbs for capture and anti-Fc-rhesus-HRP for detection^19^. The lower limit of quantification was 3.0 ng ml^−1^.

### Mathematical modeling of viral load decline after ART

After ART is initiated, viral loads in RMs typically exhibit two phases of exponential decline before becoming undetectable. To quantify these decline rates, we used a two-phase exponential decline model:

$$V\left(t\right)=A\exp \left(-{\delta }_{1}t\right)+B\exp (-{\delta }_{2}t)$$

where $V\left(t\right)$ represents the viral load at time *t*, and ${\delta }_{1}$ and ${\delta }_{2}$ are the rates of the first and the second phases of decline, respectively, which represent the death rates of short-lived and long-lived productively infected cells^41,42^. $A$ and $B$ are constants. We fitted this model to the viral load data collected at the time of ART and afterwards to estimate all the parameter values in the model. To ensure that ${\delta }_{1}$ represents the first-phase and more rapid decline, we restricted *δ*_1_ > *δ*_2_ and *A* > *B* in our fitting. We used the value of *δ*_1_ for each RM in the correlation analyses in this study.

### Immunofluorescence

To assess the population of CD20^+^, CD8α^+^ and CD4^+^ cells, we performed immunofluorescence on lymph node biopsies as described previously^43^. In brief, after heat-induced epitope retrieval (HIER) (Advanced Cell Diagnostics), antibodies were incubated overnight at room temperature: rabbit polyclonal anti-CD8 (1:1,000, HNBP2-34039, Novus), mouse monoclonal anti-CD20 (1:500, M0755, DAKO) and goat polyclonal anti-CD4 (1:1,000, AF-379-NA, R&D Systems). Slides were washed and then incubated with secondary donkey anti-goat IgG-Alexa Fluor 488, donkey anti-mouse IgG-Alexa Fluor 594 and anti-rabbit IgG-Alexa Fluor 647 (all from Molecular Probes/Thermo Fisher Scientific) for 1 h at room temperature and washed three times for 5 min in TBS + Tween (0.05% v/v). All slides were counterstained with DAPI (ready-to-use (RTU), Advanced Cell Diagnostics) for 10 min, washed and coverslipped with #1.5 GOLD SEAL cover glass (Electron Microscopy Sciences) using Prolong Gold reagent (Invitrogen). Slides were then scanned using the Akoya Fusion microscope at ×20, and image analysis was performed using QuPath software, version 0.4.3 (ref. ^44^). All BCF and TCZ were analyzed for up to three tissue sections except for folded or damaged tissue areas. Parameters used for positive cell detection were as follows: background radius 8 µm, median filter radius 0 µm, sigma 1.5 µm, minimum area 10 µm^2^, maximum area 400 µm^2^ and cell expansion 3 µm, and each threshold was adjusted from 15 to 1.5 based on intensity of each marker. Representative pictures were selected to show BCF and TCZ and were extracted using ImageJ.

### Immunophenotyping by flow cytometry

Multicolor flow cytometric analysis was performed on whole blood and lymph node mononuclear cells using predetermined optimal concentrations of the following fluorescently conjugated mAbs for the T cell panel: CD3-APC-Cy7 (clone SP34–2), Ki-67-AF700 (clone B56), HLA-DR-PerCP-Cy5.5 (clone G46–6), CCR5-APC (clone 3A9), CCR7-FITC (clone 150503), CD45RA-PE-Cy7 (clone 5H9) and CD62L-PE (clone SK11), all from BD Biosciences; CD8-BV711 (clone RPA-T8), CD4-BV650 (clone OKT4), CD95-BV605 (clone DX2) and PD-1-BV421 (clone EH12.2H7), all from BioLegend, and CD28-PE-Cy5.5 (clone CD28.2), from Beckman Coulter; and for the NK cell functional panel: CD3-APC-Cy7 (clone SP34–2), Ki-67-AF700 (clone B56), HLA-DR-PerCP-Cy5.5 (clone G46–6), CCR7-FITC (clone 150503), CD8-BV711 (clone RPA-T8); BioLegend: CD4-BV605 (clone SK3), PD-1-BV421 (clone EH12.2H7), CTLA-4-APC (clone BNI3), NKG2D-Alexa Fluor 647 (clone 1D11), CD20-BV570 (clone 2H7), TNFα-BV650 (clone Mab11), CD56-BV750 (clone 5.1H11), CD16-BV785 (clone 3G8), perforin-PE-Cy7 (clone dG9), CD14-PerCP (clone 63D3); BD Biosciences: IFNγ-PE (clone B27) and CD95-PE-Cy5 (clone DX2); R&D Systems: TIGIT-Alexa Fluor 405 (clone 741182); Invitrogen: granzyme B-eFluor 450 (clone N4TL33), CD107a-PerCP-efluor 710 (clone eBioH4A3), LAG3-efluor 506 (clone 3S223H); Beckman Coulter: CD28-PE-Cy5.5 (clone CD28.2). Flow cytometric acquisition and analysis of samples was performed on at least 1 × 10^6^ events on a Symphony A5 flow cytometer driven by the FACSDiva software package (BD Biosciences) for the T cell panel or Cytek Aurora spectral flow cytometer for the NK functional panel. Analyses of the acquired data were performed using FlowJo software, version 10.0.4.

### Intracellular cytokine staining

Cryopreserved PBMCs were thawed and rested overnight at 2–5 million cells per milliliter and then resuspended in RPMI 1640 containing 10% FBS and added to a 96-well round-bottom plate, with 1–2 million cells per well. Cells were then stimulated at 5 μg ml^−1^ with SIV_mac239_ Gag peptide pool (NIH AIDS Reagent Program) and PMA 0.1 µg ml^−1^ + ionomycin (1 µg ml^−1^) as a positive control and equimolar DMSO conditions as a negative control for 6 h; brefeldin A (1:1,000) was added to all conditions after 1 h. CD107a PerCP-eFluor710 (clone eBioH4A3) was added with stimulation conditions. After stimulation, cells were washed twice with warm PBS and stained with the following mAbs: CD3 APC-Cy7 (clone SP34-2), CD4 BV605 (clone SK3), CD8α BV711 (clone RPA-T8), CD95 PE-Cy5 (clone DX2) and CD28-PeCy5.5 (clone CD28.2). Incubation with Live/Dead Fixable Aqua was at 37 °C for 15 min; all other surface markers were incubated at 4 °C for 30 min. Cells were then washed once with FACS buffer and then fixed, permeabilized and stained with mAbs: IFNγ PE (clone B27), TNFα BV650 (clone MAb11) and IL-2 FITC (clone MQ1-17H12) at 4 °C for 30 min. Samples were washed once with FACS buffer and treated with 2% PFA for 15 min at 4 °C. After a final FACS wash, cells were resuspended in 200 µl of PBS and acquired on a five-laser Cytek Aurora using SpectroFlo software. Analyses of the acquired data were performed using FlowJo software, version 10.0.4. Cytokine frequency was quantified in CD95^+^CD8^+^ cells to exclude naive T cells^45^. Data are presented as the frequency of cytokine-expressing cells after Gag peptide stimulation after subtracting the background frequency in the negative control condition for each individual macaque and timepoint.

### Cell-associated SIV-DNA and SIV-RNA quantification

Cell-associated SIV-DNA and SIV-RNA levels were measured in approximately 1 million CD4^+^ T cells isolated from peripheral blood, bone marrow, lymph nodes and gastrointestinal tract lysed in Buffer RLT Plus (Qiagen) plus 2-mercaptoethanol. DNA and RNA were extracted using the AllPrep DNA/RNA Mini Kit (Qiagen). Quantification of SIV *gag* DNA was performed on the extracted DNA by qPCR using the 5′ nuclease (TaqMan) assay with an ABI7500 system (PerkinElmer Life Sciences). The sequence of the forward primer for SIV *gag* was 5′-GCAGAGGAGGAAATTACCCAGTAC-3′; the reverse primer sequence was 5′-CAATTTTACCCAGGCATTTAATGTT-3′; and the probe sequence was 5′-FAM (6-carboxyfluorescein)-TGTCCACCTGCCATTAAGCCCGA-TAMRA (6-carboxytetramethylrhodamine)-3′. For cell number quantification, qPCR was performed simultaneously for monkey albumin gene copy numbers. The sequence of the forward primer for albumin was 5′-TGCATGAGAAAACGCCAGTAA-3′; the reverse primer sequence was 5′-ATGGTCGCCTGTTCACCAA-3′; and the probe sequence was 5′-AGAAAGTCACCAAATGCTGCACGGAATC-3′. For cell-associated RNA quantification, RNA was reverse transcribed using Thermo Fisher Scientific’s High Capacity cDNA Reverse Transcription Kit and random hexamers. SIV *gag* and the RM *CD4* gene were quantified by qPCR of the resultant cDNA using Thermo Fisher Scientific’s TaqMan Universal Master Mix II. The CD4 primer and probe sequences were Rh-CD4-F: 5′-ACATCGTGGTGCTAGCTTTCCAGA-3′; Rh-CD4-R: 5′-AAGTGTAAAGGCGAGTGGGAAGGA-3′; and Rh-CD4-Probe: 5′-AGGCCTCCAGCACAGTCTATAAGAAAGAGG-3′. The means of two replicate wells were used in all analyses. Samples with undetectable SIV-DNA were assigned a level of 1 copy per million CD4^+^ T cells for display purposes.

### SIV quantitative viral outgrowth assay

Replication-competent SIV reservoirs were measured by the Viral Reservoir Unit of the Emory Center for AIDS Research Virology and Molecular Biomarkers Core. The frequency of latently infected cells was estimated using a limiting dilution culture assay in which CD4^+^ T cells enriched from peripheral blood, lymph node or spleen using magnetic beads and column purification (Miltenyi Biotec) were co-cultured with CEMx174 cells in five-fold serial dilutions ranging from 2 × 10^6^ cells per well to 4 × 10^5^ cells per well. The cells were cultured in RPMI 1640 containing 10% FBS and 100 U ml^−1^ IL-2 (Sigma-Aldrich). The ratio of target cells added was 4:1 for the two highest dilutions. A constant number of 1 × 10^6^ CEMx174 cells was added to all other wells. The cultures were split every 7 d, and fresh medium was added. After 21 d, the growth of virus was detected by RT–qPCR. SIV-RNA was isolated from 400 μl of the culture supernatant using the Zymo viral RNA isolation kit (Zymo Research). DNase treatment was performed using an RQ1 RNase-free DNase kit (Promega). One-step RT–qPCR targeting SIV *gag* was performed using an Applied Biosystems 7500 real-time PCR system and TaqMan Fast Virus 1-Step Master Mix (Thermo Fisher Scientific) for RT–qPCR with the following primers and probe: SIV*gag*Fwd (5′-GCAGAGGAGGAAATTACCCAGTAC-3′), SIV*gag*Rev (5′-CAATTTTACCCAGGCATTTAATGTT-3′) and SIV*gag* probe (5′-FAM-TGTCCACCTGCCATTAAGCCCGA-3IBFQ-3′). The frequencies of infected cells were determined by the maximum likelihood method and were expressed as infectious units per million (IUPM) CD4^+^ T cells.

### Statistical analysis

Statistical analyses were performed using GraphPad Prism software, version 9. No statistical methods were used to predetermine sample size. Investigators were not blinded to group allocations or when assessing outcomes. Analyses across groups were performed using two-tailed Mann–Whitney *U*-test, Kruskal–Wallis test with Dunn’s test for multiple comparisons or two-way ANOVA with Tukey’s multiple comparisons test, as appropriate. Analyses within groups were performed using two-sided Wilcoxon matched-pairs signed-rank test or Friedman test with Dunn’s test for multiple comparisons. For all statistical analyses, *P* values less than 0.05 were considered significant.

### Reporting summary

Further information on research design is available in the Nature Portfolio Reporting Summary linked to this article.

## Online content

Any methods, additional references, Nature Portfolio reporting summaries, source data, extended data, supplementary information, acknowledgements, peer review information; details of author contributions and competing interests; and statements of data and code availability are available at 10.1038/s41591-023-02570-7.

### Supplementary information

Reporting Summary

## Data Availability

Requests for data or materials described in this manuscript will be promptly reviewed by the corresponding author (A.C.) to determine if these are subject to intellectual property, confidentiality or ethical obligations. If not subject to these restrictions, data will be shared with the requester in a timely manner. Materials that can be shared will be released via a material transfer agreement. Inquiries regarding data or material availability should be directed to ann.m.chahroudi@emory.edu.
