# Supplementary material for: AZD5582 plus SIV-specific antibodies reduce lymph node viral reservoirs in antiretroviral therapy-suppressed macaques
Source: Nat Med. 2023 Oct 2;29(10):2535–46. doi: 10.1038/s41591-023-02570-7 (PMC10579098; doi:10.1038/s41591-023-02570-7)
Supplement: Supplementary file 1 — Reporting Summary [file 41591_2023_2570_MOESM1_ESM.pdf]

nature portfolio

Corresponding author(s): Ann Chahroudi

Last updated by author(s): Aug 25, 2023

Reporting Summary

Nature Portfolio wishes to improve the reproducibility of the work that we publish. This form provides structure for consistency and transparency in reporting. For further information on Nature Portfolio policies, see our Editorial Policies and the Editorial Policy Checklist.

Statistics

For all statistical analyses, confirm that the following items are present in the figure legend, table legend, main text, or Methods section.

n/a	Confirmed	
☐	☒	The exact sample size (*n*) for each experimental group/condition, given as a discrete number and unit of measurement
☐	☒	A statement on whether measurements were taken from distinct samples or whether the same sample was measured repeatedly
☐	☒	The statistical test(s) used AND whether they are one- or two-sided<br>*Only common tests should be described solely by name; describe more complex techniques in the Methods section.*
☐	☒	A description of all covariates tested
☐	☒	A description of any assumptions or corrections, such as tests of normality and adjustment for multiple comparisons
☐	☒	A full description of the statistical parameters including central tendency (e.g. means) or other basic estimates (e.g. regression coefficient) AND variation (e.g. standard deviation) or associated estimates of uncertainty (e.g. confidence intervals)
☐	☒	For null hypothesis testing, the test statistic (e.g. *F*, *t*, *r*) with confidence intervals, effect sizes, degrees of freedom and *P* value noted<br>*Give P values as exact values whenever suitable.*
☒	☐	For Bayesian analysis, information on the choice of priors and Markov chain Monte Carlo settings
☒	☐	For hierarchical and complex designs, identification of the appropriate level for tests and full reporting of outcomes
☐	☒	Estimates of effect sizes (e.g. Cohen's *d*, Pearson's *r*), indicating how they were calculated

Our web collection on statistics for biologists contains articles on many of the points above.

Software and code

Policy information about availability of computer code

Data collection	BD FACSDiva software (version 6.1.3)
Data analysis	FlowJo software (version 10), GraphPad<br>Prism v10<br>ImageJ3<br>QuPath software v0.4.3

For manuscripts utilizing custom algorithms or software that are central to the research but not yet described in published literature, software must be made available to editors and reviewers. We strongly encourage code deposition in a community repository (e.g. GitHub). See the Nature Portfolio guidelines for submitting code & software for further information.

Data

Policy information about availability of data

All manuscripts must include a data availability statement. This statement should provide the following information, where applicable:

- Accession codes, unique identifiers, or web links for publicly available datasets
- A description of any restrictions on data availability
- For clinical datasets or third party data, please ensure that the statement adheres to our policy

Requests for data or materials described in this manuscript will be promptly reviewed by the corresponding author (A.C.) to determine if these are subject to

intellectual property, confidentiality or ethical obligations. If not subject to these restrictions, data will be shared with the requester in a timely manner. Materials that can be shared will be released via a material transfer agreement. Inquiries regarding data or material availability should be directed to ann.m.chahroudi@emory.edu.

Human research participants

Policy information about studies involving human research participants and Sex and Gender in Research.

Reporting on sex and gender	N/A
Population characteristics	N/A
Recruitment	N/A
Ethics oversight	N/A

Note that full information on the approval of the study protocol must also be provided in the manuscript.

Field-specific reporting

Please select the one below that is the best fit for your research. If you are not sure, read the appropriate sections before making your selection.

- [x] Life sciences
- [ ] Behavioural & social sciences
- [ ] Ecological, evolutionary & environmental sciences

For a reference copy of the document with all sections, see nature.com/documents/nr-reporting-summary-flat.pdf

Life sciences study design

All studies must disclose on these points even when the disclosure is negative.

Sample size	Sample size calculations were based on previous data from SIV-infected ART-suppressed rhesus macaques treated with AZD5582 (Nixon et al, Nature 2020). We estimated that with a sample size of at least 7 we would be able to detect a significant difference between pre- and post-AZD5582 treatment samples in the levels of plasma RNA at the 0.05 significance level with a power of 0.90.
Data exclusions	No data were excluded from the analyses.
Replication	In vitro experiments (ICABA, ADCC, ADNP) were replicated at least twice, with similar results. RhmAb concentrations, ADA, and immune cell phenotype were measured multiple times over the course of the in vivo longitudinal experiment. Viral reservoir assessments were performed once due to limited sample availability. SIV-specific T cell responses were measured at two time points in multiple animals, one time. Immunofluorescence was performed at 3 time points per animal without further replication.
Randomization	In total, 30 Indian-origin RMs all negative for the Mamu-B*08 and -B*17 MHC class I alleles associated with an increased frequency of spontaneous control of SIV replication were selected for this research. Prior to additional interventions, groups of RMs were balanced for peak viral loads and pre-ART viral loads. Sex, Mamu-A*01 status and age at infection were also considered.
Blinding	Investigators were not blinded to group allocations or when assessing outcomes. Blinding was not considered in this study due to intervention conditions that required us to monitor animal health throughout the study.

Reporting for specific materials, systems and methods

We require information from authors about some types of materials, experimental systems and methods used in many studies. Here, indicate whether each material, system or method listed is relevant to your study. If you are not sure if a list item applies to your research, read the appropriate section before selecting a response.

Materials & experimental systems

n/a	Involved in the study	
☐	☒	Antibodies
☐	☒	Eukaryotic cell lines
☒	☐	Palaeontology and archaeology
☐	☒	Animals and other organisms
☒	☐	Clinical data
☒	☐	Dual use research of concern

Methods

n/a	Involved in the study	
☒	☐	ChIP-seq
☐	☒	Flow cytometry
☒	☐	MRI-based neuroimaging

Antibodies

Antibodies used

T cell panel:
• Live/Dead Aqua
o Fisher Scientific
o Catalog # L34966
o Clone: N/A
o Dilution: 1/150
• CD3 APC-Cy7
o Fisher Scientific
o Catalog # 557757
o Clone: SP34-2
o Dilution: 1/200
• CD4 BV650
o BioLegend
o Catalog # 317436
o Clone: OKT4
o Dilution: 1/200
• CD8� BV711
o BioLegend
o Catalog # 301044
o Clone: RPA-T8
o Dilution: 1/200
• CCR7 FITC
o Fisher Scientific
o Catalog # 561271
o Clone: 150503
o Dilution: 1/30
• CD45RA PE-Cy7
o Fisher Scientific
o Catalog # 561216
o Clone: 5H9
o Dilution: 1/100
• CD62L PE
o Fisher Scientific
o Catalog # 341012
o Clone: SK11
o Dilution: 1/20
• CD28 PeCy5.5
o Beckman Coulter
o Catalog # B24027
o Clone: CD28.2
o Dilution: 1/100
• CD95 BV605
o BioLegend
o Catalog # 305628
o Clone: DX2
o Dilution: 1/40
• CCR5 APC
o Fisher Scientific
o Catalog # BDB560748
o Clone: 3A9
o Dilution: 1/30
• PD-1 BV421
o BioLegend
o Catalog # 329920
o Clone: EH12.2H7
o Dilution: 1/40
• HLA-DR PerCP-Cy5.5
o Fisher Scientific
o Catalog # 552764
o Clone: EH12.2H7
o Dilution: 1/100
• Ki67 AF700
o Fisher Scientific
o Catalog # 561277
o Clone: B56
o Dilution:1/20

Functional Panels:
• Live/Dead Aqua
o Fisher Scientific
o Catalog # L34966
o Clone: N/A

o Dilution: 1/150
• CCR7 FITC
o Fisher Scientific
o Catalog # 561271
o Clone: 150503
o Dilution:1/30
• CD3 APC-Cy7
o Fisher Scientific
o Catalog # 557757
o Clone: SP34-2
o Dilution: 1/200
• CD4 BV605
o BioLegend
o Catalog # 344646
o Clone: SK3
o Dilution: 1/100
• CD8⍰ BV711
o BioLegend
o Catalog # 301044
o Clone: RPA-T8
o Dilution: 1/100
• CD14 PerCP
o BioLegend
o Catalog # 367152
o Clone: 63D3
o Dilution: 1/100
• CD20 BV570
o BioLegend
o Catalog # 302332
o Clone: 2H7
o Dilution: 1/100
• CD16 BV785
o BioLegend
o Catalog # 302046
o Clone: 3G8
o Dilution: 1/100
• CD56 BV750
o BioLegend
o Catalog # 362556
o Clone: 5.1H11
o Dilution: 1/100
• NKG2D AF647
o BioLegend
o Catalog # 320826
o Clone: 1D11
o Dilution: 1/100
• CD95 PeCy5
o BD
o Catalog # 559773
o Clone: DX2
o Dilution:1/100
• CD28 Pe-Cy5.5
o Beckman Coulter
o Catalog # B24027
o Clone: CD28.2
o Dilution: 1/40
• PD-1 BV421
o BioLegend
o Catalog # 329920
o Clone: EH12.2H7
o Dilution: 1/40
• HLA-DR PerCP/Cy5.5
o Fisher Scientific
o Catalog # 552764
o Clone: G46-6
o Dilution: 1/80
• Ki67 AF700
o Fisher Scientific
o Catalog # 561277
o Clone: B56
o Dilution: 1/30
• CD107a PerCP eFluor710
o Thermo Scientific
o Catalog # 46-1079-42
o Clone: eBioH4A3
o Dilution: 1/100

• TNFa BV650
o BioLegend
o Catalog # 502938
o Clone: MAb11
o Dilution: 1/30
• Granzyme B eFluor450
o eBioscience
o Catalog # 48-8896-42
o Clone: N4TL33
o Dilution: 1/30
• IFNg PE
o BD Biosciences
o Catalog # 554701
o Clone: B27
o Dilution: 1/10
• Perforin PE-Cy7
o BioLegend
o Catalog # 308126
o Clone: dG9
o Dilution: 1/30
• IL-2 FITC
o BD Pharmingen
o Catalog: # 55465
o Clone: MQ1-17H12
o Dilution: 1/10

IHC
• CD8
o Novus
o Catalog # HNBP2-34039
o Clone: N/A
o Dilution: 1/1000
• CD20
o DAKO
o Catalog # M0755
o Clone: L26
o Dilution: 1/500
• CD4
o R&D Systems
o Catalog # AF-379-NA
o Clone: EPR6855
o Dilution: 1/1000
• Donkey anti-goat-Alexa488
o Molecular Probes/ThermoFisher Scientific
o Catalog # A-11055
o Clone: N/A
o Dilution: 1/500
• Donkey anti-mouse IgG-Alexa 594
o Molecular Probes/ThermoFisher Scientific
o Catalog # A-21203
o Clone: N/A
o Dilution: 1/500
• Donkey anti-Rabbit IgG-Alexa 647
o Molecular Probes/ThermoFisher Scientific
o Catalog # A-31573
o Clone: N/A
o Dilution: 1/500

ICABA
• Anti-p27-FITC antibody
o WNPRC Immunology Services
o Catalog # N/A
o Clone: N/A
o Dilution: 1/500
• Goat anti-human Ig Fc-PE
o eBioscience
o Catalog # 12-4998-82
o Clone: Polyclonal
o Dilution: 1/400

Compensation Beads
• UltraComp eBeads Compensation Beads
o Thermo Fisher Scientific
o Catalog # 01-2222-42
• ArcTM live/dead reactive beads (comps)
o BD Biosciences

o Catalog # A10346

For PK/ADA assay: ITS09.01-LS, ITS102.01-LS, ITS103.01-LS, ITS113.01-LS , anti-ITS09, anti-ITS102, anti-ITS103, and anti-ITS113 provided by Mario Roederer's group at the NIH.

Validation	The specificity of the antibodies purchased from commercial sources (BD Biosciences, Biolegend, and Beckman Coulter) were validated by the manufacturer as noted on their websites (links provided above for each antibody). Validation was also confirmed using FMO controls and known expression patterns on lymphocyte subsets. We also used our previous published data as a validation method.<br>1. Nixon, C. C. et al. Systemic HIV and SIV latency reversal via non-canonical NF-kappaB signaling in vivo. Nature 578, 160-165 (2020). https://doi.org:10.1038/s41586-020-1951-3<br>2. Pollara, J. et al. Functional Homology for Antibody-Dependent Phagocytosis Across Humans and Rhesus Macaques. Front Immunol 12, 678511 (2021).

Eukaryotic cell lines

Policy information about cell lines and Sex and Gender in Research

Cell line source(s)	A66 cell line: provided by James Hoxie, University of Pennsylvania, Philadelphia, PA<br>CEM174: NIH AIDS Reagent Repository
Authentication	Cell lines were authenticated by morphological identification and virus susceptibility profiles.
Mycoplasma contamination	Cell lines were tested negative for mycoplasma by the supplier
Commonly misidentified lines (See ICLAC register)	No commonly misidentified cell lines were used

Animals and other research organisms

Policy information about studies involving animals; ARRIVE guidelines recommended for reporting animal research, and Sex and Gender in Research

Laboratory animals	Thirty male and female (25 males and five females) Indian RMs, two to five years of age, with the exclusion of Mamu B*08 and B*17 positive animals, were enrolled in this study
Wild animals	The study did not involve wild animals.
Reporting on sex	25 male and five female Indian RMs were selected for this study. Selection criteria for this study was availability of animals.
Field-collected samples	This study did not involve samples collected from the field..
Ethics oversight	RMs infected with SIV were housed at the Emory National Primate Research Center (Atlanta, GA) and treated according to Emory University and Emory National Primate Research Center regulations. Animal care facilities are accredited by the U.S. Department of Agriculture (USDA) and the Association for Assessment and Accreditation of Laboratory Animal Care (AAALAC) International. The Emory University IACUC approved this study (PROTO201700286).

Note that full information on the approval of the study protocol must also be provided in the manuscript.

Flow Cytometry

Plots

Confirm that:

☒ The axis labels state the marker and fluorochrome used (e.g. CD4-FITC).

☒ The axis scales are clearly visible. Include numbers along axes only for bottom left plot of group (a 'group' is an analysis of identical markers).

☒ All plots are contour plots with outliers or pseudocolor plots.

☒ A numerical value for number of cells or percentage (with statistics) is provided.

Methodology

Sample preparation	EDTA-anticoagulated blood samples were collected regularly and whole blood was used for flow cytometry. In addition, fine needle aspirations (FNA) of lymph nodes and lymph node biopsies were collected. FNA samples were washed and cells isolated. After two washes in RPMI and removal of connective and fat tissues, lymph node biopsies were ground using a 70-µm cell strainer and then washed and filtered.
Instrument	Symphony A5 flow cytometer (BD Biosciences), Cytek Aurora spectral flow cytometer
Software	Analyses of the acquired data were performed using FlowJo™ software (Tree Star, version 10.0.4).

Cell population abundance	Flow cytometric sorting of cells was not performed in this study.
Gating strategy	For basic immunophenotyping, stained cells were gated on singlets, then lymphocytes, then live CD3+ cells. CD4+ or CD8+ T cells were then gated on for further phenotyping. NK cells were gated on using live CD3- cell gating or live CD3-NKGD2D+ cell gating.<br><br>The gating strategy to detect SIV-infected A66 cells using ITS antibodies is shown in Extended Data Figure 1.<br><br>The gating strategy to identify SIV-specific CD8+ memory T cells is shown in Extended Data Figure 6.

☒ Tick this box to confirm that a figure exemplifying the gating strategy is provided in the Supplementary Information.
